# The role of mothers and fathers in children’s health care use

**DOI:** 10.1007/s10754-026-09417-x

**Published:** 2026-06-29

**Authors:** Toni Mora, Teresa Bago d’Uva, Pilar García-Gómez, Manuel Flores

**Affiliations:** 1 Research Institute for Evaluation and Public Policies (IRAPP), Universitat Internacional de Catalunya (UIC), Barcelona, Spain; 2 Department of Applied Economics, Erasmus School of Economics, Rotterdam, The Netherlands; 3 Serra Húnter Fellow, Department of Applied Economics, Universitat Autònoma de Barcelona, Barcelona, Spain

**Keywords:** Health care use, Intergenerational associations, SES-health gradient, Child fixed effects, Longitudinal medical records, I10, I14, I18, J13

## Abstract

We use rich longitudinal administrative medical records to examine the separate roles of mothers and fathers in children’s health care use. We do so distinguishing between different types of care and child sex. Overall, we find limited evidence of an association between parents’ education and their children’s health care use. Mothers’ health care use appears in general more strongly associated than fathers’ health care use with that of their children. On the other hand, for some types of care (GP and specialist visits), these associations in health care use are stronger within the same-sex parent–child dyads. These results are not (fully) explained by parental nor children’s health needs and health behaviors and are robust to the inclusion of GP fixed effects. These associations remain after controlling for unobserved time-invariant factors suggesting that non-health related family shocks also play a role. Our findings have potentially important policy implications, as patterns of health care seeking are established early in life, which the literature shows have long-lasting effects over people’s life course.

**Supplementary Information:**

The online version contains supplementary material available at https://doi.org/10.1007/s10754-026-09417-x.

## Introduction

Poor health in childhood has long-lasting negative effects on health and socioeconomic status later in life (Almond & Currie, 2011; Almond et al., 2018; Flores & Wolfe, 2023; Montez & Hayward, 2014; Smith, 2009). Ensuring that all children have access to adequate medical care has therefore been of great policy concern for decades (Wolfe, 1980)., This issue has received a great deal of attention especially in the United States (US) where, despite important expansions in public health insurance for (low-income) children since the 1980 s (Currie et al., 2008), 5.1% of all children under 18 years (10.3% of the total population) remained without health insurance in 2019 (NCHS 2020). However, even in countries with generous and (quasi)universal health care coverage systems, equal access to health care is not guaranteed. For instance, considerable socioeconomic-related inequalities in children’s health care use have been documented in Germany (Reinhold & Jürges, 2012), the United Kingdom (Petrou & Kupek, 2005) and Norway (Olsen et al., 2021). Inadequate health care during childhood may have detrimental effects not only on children’s current health but also on their education and subsequent later life health and labor market outcomes (Cohodes et al., 2016; Miller & Wherry, 2019; Wüst, 2022). This may be exacerbated by intergenerational transmission of patterns of health care use.

This paper contributes to the evidence on socioeconomic inequalities in health care use and its intergenerational transmission in several ways. We are able to distinguish between types of health care and child sex, and to study the separate role of fathers and mothers in their children’s health care use. Additionally, we use rich longitudinal administrative medical records. These include information on health and health care use for children and their parents linked with parental socioeconomic status from a large municipality in a country (Spain) with a statutory quasi-universal health care system. In our empirical analyses, we estimate associations between maternal and paternal socioeconomic status (in particular, education level) and health care use of their children. While previous studies tend to find a larger role of mother’s education compared to that of fathers, few investigate this separately for daughters and sons. Doing so is important to establish whether those associations indeed differ predominantly by parent sex, or rather by same-sex parent–child combinations, as has been shown for the intergenerational transmission of health-risk behaviors (Wickrama et al., 1999). We also go beyond several previous studies in being able to control extensively for child and parental health and lifestyle variables. This is relevant because education may contribute to higher cognition and subsequently improve decision-making patterns in general (Cutler & Lleras-Muney, 2010). We are therefore interested in the implications that this might have for decisions that parents take regarding health care use of their children. In addition, we investigate the association between maternal and paternal and children’s health care use by type of health care use. Finally, we assess the extent to which those associations are explained by time-invariant characteristics, like parental preferences, by controlling for children fixed effects (following the estimation method proposed by Correia et al. (2020)). The resulting associations are informative about the role of family shocks, other than changes in parental and child health, as potential mechanisms linking parental and children’s health care use.

Much of the evidence on the determinants of children’s HCU comes from medical studies.^1^ Drawing overall conclusions from this literature is difficult, as results may be partly driven by methodological differences, such as the choice of parental characteristics, types of health care use covered or the use of a particular empirical method. The richness of our data coupled with our empirical strategy make it possible to tackle a number of limitations of previous studies and so expand them in three broad directions.

First, while earlier research on the predictors of children’s health care use has typically focused on maternal or overall parental characteristics, much less is known about the role of fathers. Our results show that this is indeed also important. Namely, we find that maternal education is positively associated with the number of diagnostic tests, while the number of emergency visits is only associated, and negatively, with paternal education. Additionally, we observe transmission of health care use from both mothers and fathers to their children. These results are in line with recent evidence on intergenerational transmission of health showing that both paternal and maternal birth weights significantly predict health at birth (Giuntella et al., 2023).

Second, only a minority of studies of children’s health care use have distinguished between different types of health care (e.g., primary care, specialist, and emergency room visits), and even fewer have made that distinction both for children’s own health care use and for their parents’. As a whole, and to the best of our knowledge, no previous study has investigated the intergenerational transmission of different types of health care use from both mothers and fathers to their children, distinguishing as well by the child’s sex. Our results show the importance of these distinctions – namely, intergenerational transmission is observed mainly within the same type of care, and often within the same-sex parent–child dyads. For example, while mothers’ health care use is in general more strongly associated with that of their children, for specialist visits, and – depending on the specification – for GP and ER visits, we find evidence of intergenerational transmission in health care use within the same-sex parent–child dyads. Gaining insights on these issues is important to be able to inform health policy makers on more effective and efficient ways to address (socioeconomic) inequalities in children’s health care use.

Finally, and to the best of our knowledge, this is the first study that analyzes child’s health care use controlling extensively for child and parental health status and behaviors, time-invariant unobserved factors, as well as supply factors. None of the previous longitudinal studies of the association between parental socioeconomic status and children’s health care use (Saxena et al., 1999, Petrou & Kupek, 2005, and Olsen et al., 2021) accounts for children’s health (i.e., their need for health care) or for parental lifestyles, behaviors, and own health care use in their analyses. This is important as, for instance, different childhood health conditions and their severity are major predictors of the extent to which children use different health care services (Berra et al., 2009; Newacheck, 1992). Furthermore, the few longitudinal studies examining the associations between parental and children’s health care use (Janicke et al., 2001; Riley et al., 1993) relied on self-reported data from surveys, relatively small samples, and short follow-up periods. In addition, they often omit supply factors such as distance to health care providers or GP fixed effects. Finally, we also include child fixed effects (FEs), which has not been previously done in analyses of health care use of children, even those using longitudinal datasets. This makes it possible to account for any time-invariant unobserved factors, such as parents’ preferences for certain types of health care. Comparisons of estimates including and excluding child FEs are informative about the role of time-variant (e.g. family shocks such as parental job loss) versus time-invariant (e.g. parental preferences) as potential drivers of the association between health care use of parents and their children.

Our results thus show evidence of intergenerational transmission from mothers’/fathers’ use of a *certain* type of health care to their sons’ and daughters’ (also controlling for parental and children’s health and other characteristics, and for child FEs). Most of children’s health care use in general is likely decided by their parents. Nevertheless, to the extent that patterns of care seeking are established early in life (Osborne et al., 1989, Walker & Greene, 1989, Whitehead et al., 1986), our results are consistent with one of the mechanisms behind the recently documented intergenerational transmission of overall health (Andersen, 2021; Bencsik et al., 2023; Halliday et al., 2021).

The rest of the paper is organized as follows. The next section summarizes the existing evidence on the role of parental characteristics in children’s health care use, to which this paper further contributes. Sect. "Data, institutional background and conceptual framework" describes the institutional background of our application and our data. Sect. "Econometric model" illustrates the empirical model used in the analyses and Sect. "Results" discusses the results obtained. The last section summarizes the main findings and concludes.

## Previous studies

### Parental socioeconomic status and children’s health care use

Earlier studies show mixed evidence on the association between mother’s and father’s education and their children’s health care use. For instance, in the US, maternal education has been shown to predict low use but not high use of doctor services among children (Newacheck, 1992), doctor visits but not hospitalizations among children with special health care needs (Aday et al., 1993), nor children’s number of visits to primary care pediatricians (Janicke et al., 2001). In another US study, parent’s education level was associated with children’s receipt of recommended routine care (Hughes & Wingard, 2008). In a study covering 11 European countries, parental education was shown to predict children’s probability of using health care but not the intensity of use (Berra et al., 2009). A recent national cohort study covering all Norwegian children aged 1–16 years in the period 2008–2016 found that those of parents with a high educational level had the lowest admission probability to inpatient hospital care and number of admissions, but the highest cost per admission (Olsen et al., 2021).^2^

Relatedly, a few studies (in the UK) have examined associations between parental social class (based on occupations) and children’s HCU. In a large national cohort study in England and Wales (Saxena et al., 1999), children from the bottom social class had a higher number of general practitioner (GP) visits, for any level of severity, and more home visits than children from the top social class. Instead, no significant differences were found in the number of consultations for preventive activities. In another study that followed a large cohort of children born in two counties of Southern England during their first 10 years of life (Petrou & Kupek, 2005) children born into lower social classes (classes II, III-NM, III-M, IV, and V) were more likely to be admitted to hospital, spent longer in hospital overall, and generated greater hospital costs than children born into the highest social class (class I). It is important to note neither Saxena et al. (1999), Petrou and Kupek (2005) nor Olsen et al. (2021) control for children’s health (i.e., their need for health care). This is important as different childhood health conditions and their severity are major predictors of the extent to which children use different health care services (Berra et al., 2009; Newacheck, 1992) and they may be correlated with parental social class.^3^

### Parental health care use and children’s health care use

Earlier studies also provide mixed evidence concerning the associations between parental health care use and that of their children and, to our knowledge, only a minority have distinguished between different types of health care and none of them has considered health care use of both fathers and mothers. For instance, US studies have shown that maternal health care use predicts that of their children within the same year (e.g., Newacheck, 1992) as well as in the following two years (Riley et al., 1993). Instead, Wolfe (1980) found only limited evidence of an association between doctor visits of parents and children, but a significantly negative and positive association for private practitioners and emergency room (ER) visits, respectively. Janicke et al. (2001) found also limited evidence of an association between mothers’ and children’s health care use.

### Parental health and lifestyle and children’s health care use

Finally, concerning the associations of parental health and health behaviors with children’s HCU, previous studies show also mixed results. For instance, Riley et al. (1993) found no evidence of an association between maternal health and children’s total visits (acute and preventive) to a medical plan in the US. On the other hand, Minkovitz et al. (2005) did find a higher use of acute care at 30–33 months and a lower receipt of preventive services, including age-appropriate well-child visits and up-to-date vaccinations at 24 months for infectious diseases, among children whose mothers had depressive symptoms at 2–4 months. Maternal worries about children’s health have been also shown to be a major predictor of children’s number of visits to primary care pediatricians (Janicke et al., 2001) and parents’ beliefs about the timing of routine checkups were strongly associated with their children’s receipt of recommended routine care (Hughes & Wingard, 2008), both in the US.

### Contributions of this paper

We overcome a number of limitations of earlier studies on children’s determinants of health care use. Firstly, they have typically relied on self-reported data from surveys^4^ (Aday et al., 1993; Barros et al., 2012; Berra et al., 2009; Hughes & Wingard, 2008; Janicke et al., 2001; Minkovitz et al., 2005; Newacheck, 1992; Riley et al., 1993; Saxena et al., 1999; Victora et al., 2003, 2012; Wolfe, 1980), often with small samples (Hughes & Wingard, 2008; Janicke et al., 2001; Wolfe, 1980) and/or with low response rates (Berra et al., 2009; Janicke et al., 2001; Minkovitz et al., 2005; Riley et al., 1993) which may cause further sample selection associated with relevant factors and so selection bias.^5^ They also often come from a single cross-section (Aday et al., 1993; Berra et al., 2009; Hughes & Wingard, 2008; Newacheck, 1992; Wolfe, 1980) with a few exceptions using longitudinal data (Janicke et al., 2001; Minkovitz et al., 2005; Olsen et al., 2021; Petrou & Kupek, 2005; Saxena et al., 1999) or retrospective data (Riley et al., 1993), but not always accounting for the longitudinal feature of the data in the analysis and so not controlling for time-invariant unobserved heterogeneity that may capture parents’ preferences for health care. Existing studies also typically pay little or no attention to the role of fathers (Aday et al., 1993; Barros et al., 2012; Berra et al., 2009; Hughes & Wingard, 2008; Janicke et al., 2001; Minkovitz et al., 2005; Newacheck, 1992; Petrou & Kupek, 2005; Riley et al., 1993; Saxena et al., 1999; Victora et al., 2003, 2012; Wolfe, 1980) and also do not make a distinction between specific types of medical care use by children (Berra et al., 2009; Janicke et al., 2001; Newacheck, 1992; Olsen et al., 2021; Riley et al., 1993) and parents (Aday et al., 1993; Berra et al., 2009; Janicke et al., 2001; Minkovitz et al., 2005; Olsen et al., 2021; Riley et al., 1993; Saxena et al., 1999; Wolfe, 1980), and sometimes omit young children (Berra et al., 2009; Janicke et al., 2001; Riley et al., 1993). Another important aspect that is typically not considered is the role of supply-side factors such as the distance to different health care providers and GP fixed effects (Aday et al., 1993; Barros et al., 2012; Berra et al., 2009; Hughes & Wingard, 2008; Janicke et al., 2001; Minkovitz et al., 2005; Newacheck, 1992; Olsen et al., 2021; Petrou & Kupek, 2005; Riley et al., 1993; Saxena et al., 1999; Victora et al., 2003, 2012; Wolfe, 1980). In addition, and to the best of our knowledge, none of these studies has investigated if the potential intergenerational transmission in health care use is stronger within the same-sex parent–child dyads. Finally, as mentioned earlier, previous studies have not controlled for child FEs to investigate the extent to which correlations found between, for example, parental and child health care use are driven by correlated time-invariant unobserved factors such as parental attitudes and preferences for (different types of) health care use. To the best of our knowledge, this is the first study to tackle all these issues.^6^

## Data, institutional background and conceptual framework

### Institutional background

The Spanish National Health Service (SNHS) provides universal coverage and is funded from taxes since the Health Care General Act was implemented in 1986. This replaced a system based on social contributions and extended health care coverage to virtually the entire population (García-Armesto et al., 2010). The SNHS provides generous health care coverage to all Spanish residents (civil servants can chose between coverage in the SNHS or in MUFACE, *Mutualidad General de Funcionarios Civiles del Estado*, a special health insurance scheme). Health care is free at the point of use except for a non-refundable coinsurance rate for outpatient prescription pharmaceuticals, which has been 40% of the retail price since the early 1980s. Moreover, and importantly for this paper, the SNHS is characterized by strong primary care gatekeeping. General practitioners (GPs) act as the main entry point to the system, and referrals are typically required for access to specialist care and diagnostic tests, whereas emergency services can be accessed directly without referral.

Health expenditure in Spain rose from 3,130 to 3,293 US$ per capita (PPP-adjusted 2015 US$) between 2010 and 2019, remaining stable at 9.1% of GDP. Most of this health expenditure is financed by the public sector (72% during 2010–2019), sourced mainly from taxation (OECD, 2022a). Despite having a lower level of health spending per capita than the OECD average (88% of the OECD average during 2010–2019, OECD, 2022a), Spain ranks relatively well in several health and health care dimensions such as life expectancy, cause-specific mortality rates, avoidable mortality, immunization coverage in children, but below average in dimensions such as low birth weight and waiting times for certain surgeries (OECD, 2022b, c).

### Conceptual framework and expected gradients

The institutional features described above provide a useful framework for interpreting potential differences in intergenerational associations and socioeconomic gradients across types of health care utilization. First, for GP visits, access barriers are relatively low, and utilization partly reflects health-related preferences and care-seeking behavior. Intergenerational associations in GP use may therefore reflect the transmission of health behaviors, attitudes toward health care, and knowledge about navigating the health care system. Socioeconomic gradients may arise from differences in health literacy, time constraints, and parental investments in children’s health. Second, for specialist visits and diagnostic tests, access is more strongly mediated by the primary care physician through the referral process. Utilization is therefore expected to be more closely linked to medical need and professional decision-making, potentially leading to weaker socioeconomic gradients and weaker intergenerational associations than for GP visits. Nevertheless, parental involvement and communication with health professionals may still influence referral decisions and follow-up care. Finally, emergency care represents a distinct category because it can be accessed without referral and often reflects acute health events. At the same time, emergency utilization may also capture differences in parental constraints, such as difficulties in accessing primary care during working hours, as well as differences in perceptions of urgency. Intergenerational associations in emergency care may therefore reflect both medical need and parental decision-making.

Another useful distinction in this context is between planned and unplanned care. Specialist visits, diagnostic tests, and, to some extent, GP visits usually involve planned interactions with the health care system, whereas emergency visits typically reflect unplanned episodes of care. Intergenerational associations and socioeconomic gradients may be expected to be stronger for planned care, where parental knowledge, preferences, and resources play a larger role in utilization decisions, and weaker where utilization is primarily driven by acute medical need.

Disaggregating parental health care utilization by type of service also helps to clarify the mechanisms underlying intergenerational associations. If parent–child correlations primarily reflect similarities in health status or disease-specific risks, stronger associations would be expected within the same service types. Cross-type associations, in contrast, may capture broader patterns of health care–seeking behavior, information transmission, and parental preferences for using the health care system. Because different services vary in the degree of discretion involved in utilization decisions, associations may be stronger for services such as GP visits or emergency care, where parental behavior and constraints play a larger role, than for services that depend more heavily on physician referral. Examining utilization separately by type of service therefore provides additional insight compared with an aggregated measure of parental health care use.

Several mechanisms may generate correlations between parental and child health care utilization even after controlling for observed health and socioeconomic characteristics (Almond & Currie, 2011; Cutler & Lleras-Muney, 2010). Positive associations may arise through the transmission of health-related preferences, information about navigating the health care system, or parental investments in child health. Time constraints and competing demands within the household may also link parental and child utilization decisions. In addition, common shocks affecting the household or the local health care environment—such as changes in employment conditions, congestion in health centers, or variation in provider practice styles—may simultaneously affect the utilization of parents and children. At the same time, some mechanisms may generate weaker or even negative associations. For example, time constraints or substitution in the use of health services within the household may reduce children’s utilization when parental utilization increases. Because these mechanisms may operate simultaneously and sometimes in opposite directions, the sign and magnitude of intergenerational associations are ultimately empirical questions.

Differences in intergenerational associations may also arise by the sex of parents and children. In many households, mothers play a more prominent role in organizing children’s health care and accompanying children to medical visits, which may lead to stronger associations between maternal utilization and children’s utilization than between paternal utilization and children’s utilization. Differences between sons and daughters may also reflect variation in parental role modeling or communication about health behaviors, which may be stronger within same-sex parent–child pairs.

These considerations provide ex ante expectations that help interpret the empirical results presented below, particularly differences in intergenerational associations across types of health care use and across parent–child pairs.

### Data

We use rich individual-level longitudinal data from administrative records of patients followed over up to seven years (2004–2010) in six primary care centers and two hospitals in the municipality of Badalona (north-east of Barcelona, Spain), which provide care to a population of around 110,000 individuals.^7^ This municipality is fairly representative of the Catalan and Spanish population in terms of socioeconomic status. For example, its gross per capita household income level between 2010 and 2018 was about 6 percentage points below the Catalan average (Idescat, 2021) and about 6 percentage points above the Spanish average (INE, 2021). The dataset contains 103,175 individuals, which is 94% of the targeted population. This contains all individuals with at least one contact with the public health care system, at either one of the included primary care centers and/or reference hospitals, between 1 January 2004 and 31 December 2010. The remaining 6% either did not visit any healthcare providers during this period, or relied only on the private health care system, or were transferred or moved to other centers before their first visit during our sample period. Our analysis focus on children and their parents. As is common in studies based on public medical records, our data excludes children who relied *only* on the private health care system during the reference period. Nevertheless, the fact we observe 94% of the overall population suggests that this excludes a small share of the children in our setting.

We link children to their parents via household identifiers provided by Idescat (the Catalan Institute of Statistics) and make use of comprehensive information on health care use of children, as well as of their fathers and mothers. Our dataset includes the number of visits to the GP, specialist and ER, the number of hospitalizations and bed days, as well as children’s number of laboratory, radiology and other diagnostic tests aggregated at the yearly level. In addition, the dataset includes detailed health diagnoses of chronic and severe conditions for both children and parents, as well as functional limitations, clinical measurements of weight and height,^8^ health behaviors of parents (smoking and drinking) and date of hospital admission and discharge. Finally, the data also contains information on each patient’s age, sex, place of birth and address.^9^

We further link this data by means of a unique individual identifier with the Population Census to retrieve information on other parental socioeconomic characteristics (for both mothers and fathers) such as education level, employment and occupation. This information comes from the 2001 Census. Because Spain experienced an immigration boom from the mid-1990s until 2006, particularly in the early 2000 s (Bentolila et al., 2008), the immigrant population is underrepresented in our sample. According to the population Census of 2001 and 2011, the percentage of immigrants rose from about 4% to 11% in Spain and from about 3% to 14% in the municipality of Badalona. Linking our administrative medical records with Census data causes a large drop in the share of immigrant children from 11 to 1%. Our final sample is still representative of the native population.

Our final sample is an unbalanced panel of 93,365 annual observations of 15,292 children younger than 18 years old with information on all variables used in the analyses. The panel is unbalanced because some children age out (children are followed while they are under 18 years old). Table 1 shows descriptive statistics and detailed information on each of the variables included in our empirical analyses for the full sample including all children, as well as sub-samples of daughters and sons.

**Table 1:** Descriptive statistics

	All children		Daughters		Sons	
	Mean	SD	Mean	SD	Mean	SD
*Child health care use*						
GP visits	6.571	8.312	6.536	8.128	6.603	8.481
Specialist visits	0.444	1.262	0.436	1.266	0.450	1.258
Diagnostic tests	0.306	0.760	0.323	0.784	0.289	0.737
Emergency room (ER) visits	0.117	0.426	0.108	0.420	0.124	0.431
*Parental education and income*						
Fathers' schooling years	9.670	4.040	9.647	3.970	9.690	4.104
Mothers' schooling years	9.763	4.137	9.728	4.096	9.796	4.175
Father primary education (0–1)	0.195	0.396	0.188	0.391	0.201	0.401
Father secondary education (0–1)	0.647	0.478	0.661	0.473	0.635	0.481
Father tertiary education (0–1)	0.158	0.364	0.151	0.358	0.164	0.370
Mother primary education (0–1)	0.192	0.394	0.190	0.392	0.194	0.395
Mother secondary education (0–1)	0.625	0.484	0.632	0.482	0.618	0.486
Mother tertiary education (0–1)	0.184	0.387	0.179	0.383	0.189	0.391
Household labor income (in 1000 €)	36.315	22.593	36.293	22.380	36.336	22.792
*Parental health care use*						
Fathers' visits to GP	8.422	8.438	8.430	8.348	8.413	8.522
Mothers' visits to GP	10.469	9.157	10.520	9.187	10.422	9.130
Fathers' visits to specialist	3.354	4.565	3.367	4.463	3.342	4.658
Mothers' visits to specialist	3.690	5.085	3.686	5.048	3.694	5.121
Fathers' ER visits	0.681	0.984	0.692	0.996	0.671	0.972
Mothers' ER visits	0.696	0.998	0.705	1.008	0.688	0.989
*Child health and lifestyle*						
Children's number of comorbidities	2.030	2.313	2.041	2.311	2.020	2.315
Children with CVD (0–1)	0.015	0.122	0.015	0.121	0.015	0.122
Children with asthma/pulmonary problems (0–1)	0.070	0.255	0.061	0.239	0.078	0.269
Children with any mental disorder (0–1)	0.003	0.054	0.003	0.057	0.003	0.052
Child normal or underweight (0–1)	0.685	0.464	0.681	0.466	0.689	0.463
Child overweight (0–1)	0.118	0.323	0.117	0.322	0.119	0.324
Child obese (0–1)	0.039	0.193	0.040	0.196	0.038	0.190
Child BMI not measured (0–1)	0.158	0.364	0.162	0.368	0.154	0.361
*Parental health and lifestyle*						
Parental number of comorbidities	6.605	3.622	6.628	3.634	6.583	3.610
Fathers' hospitalization days	0.513	2.242	0.538	2.289	0.489	2.197
Mothers' hospitalization days	0.341	1.532	0.352	1.603	0.330	1.462
Any parent with mental disorder (0–1)	0.186	0.389	0.188	0.391	0.185	0.388
Any parent passed away (0–1)	0.016	0.126	0.017	0.128	0.016	0.125
Parental BMI not measured (0–1)	0.370	0.483	0.364	0.481	0.375	0.484
Both parents with normal weight (0–1)	0.081	0.272	0.081	0.273	0.080	0.272
At least one parent is overweight (0–1)	0.257	0.437	0.262	0.440	0.253	0.435
At least one parent is obese (0–1)	0.292	0.455	0.293	0.455	0.291	0.454
At least one parent smoker and/or heavy drinker (0–1)	0.427	0.495	0.434	0.496	0.422	0.494
*Other controls*						
Child is female (0–1)	0.484	0.500				
Child's age	7.578	5.668	7.622	5.664	7.537	5.671
Child has immigrant status (0–1)	0.006	0.076	0.006	0.077	0.006	0.075
Fathers' age	43.732	6.155	43.747	6.119	43.719	6.188
Mothers' age	42.232	6.389	42.238	6.360	42.226	6.416
Minimum distance to GP in km	1.722	5.105	1.772	5.422	1.676	4.789
Minimum distance to hospital in km	3.131	5.363	3.190	5.672	3.076	5.056
Year = 2004 (0–1)	0.163	0.369	0.163	0.369	0.163	0.370
Year = 2005 (0–1)	0.156	0.363	0.156	0.363	0.156	0.363
Year = 2006 (0–1)	0.149	0.356	0.150	0.357	0.149	0.356
Year = 2007 (0–1)	0.143	0.350	0.143	0.350	0.143	0.350
Year = 2008 (0–1)	0.136	0.343	0.136	0.343	0.136	0.343
Year = 2009 (0–1)	0.130	0.336	0.129	0.336	0.130	0.336
Year = 2010 (0–1)	0.123	0.328	0.123	0.328	0.123	0.328
N. of children	15292		7387		7905	
N. of observations	93365		45174		48191	

## Econometric model

Our empirical model for children’s health care use (HCU) relies on the so-called behavioral model of health services use (Aday & Andersen, 1981; Andersen, 1995) and is specified as follows:

1 $$\begin{aligned}E\left({HCU}_{c,t,k}|{{\boldsymbol{x}}}_{{\boldsymbol{c}},{\boldsymbol{t}}}\right)&=\mathrm{exp}\big({\alpha}_{0}+{{\boldsymbol{M}}{\boldsymbol{E}}{\boldsymbol{d}}{\boldsymbol{u}}{\boldsymbol{c}}}_{{\boldsymbol{c}},{\boldsymbol{t}}}^{\boldsymbol{^{\prime}}}{{\boldsymbol{\alpha}}}_{1}\\&+{{\boldsymbol{F}}{\boldsymbol{E}}{\boldsymbol{d}}{\boldsymbol{u}}{\boldsymbol{c}}}_{{\boldsymbol{c}},{\boldsymbol{t}}}^{\boldsymbol{^{\prime}}}{{\boldsymbol{\alpha}}}_{2}+{\alpha}_{3}{Inc}_{c,t}\\&+{{\boldsymbol{M}}{\boldsymbol{H}}{\boldsymbol{C}}{\boldsymbol{U}}}_{{\boldsymbol{c}},{\boldsymbol{t}}}^{\boldsymbol{^{\prime}}}{{\boldsymbol{\alpha}}}_{4}+{{\boldsymbol{F}}{\boldsymbol{H}}{\boldsymbol{C}}{\boldsymbol{U}}}_{{\boldsymbol{c}},{\boldsymbol{t}}}^{\boldsymbol{^{\prime}}}{{\boldsymbol{\alpha}}}_{5}\\&+{{\boldsymbol{C}}{\boldsymbol{H}}}_{{\boldsymbol{c}},{\boldsymbol{t}}}^{\boldsymbol{^{\prime}}}{{\boldsymbol{\alpha}}}_{6}+{{\boldsymbol{P}}{\boldsymbol{H}}}_{{\boldsymbol{c}},{\boldsymbol{t}}}^{\boldsymbol{^{\prime}}}{{\boldsymbol{\alpha}}}_{7}\\&+{{\boldsymbol{C}}{\boldsymbol{H}}{\boldsymbol{B}}}_{{\boldsymbol{c}},{\boldsymbol{t}}}^{\boldsymbol{^{\prime}}}{{\boldsymbol{\alpha}}}_{8}+{{\boldsymbol{P}}{\boldsymbol{H}}{\boldsymbol{B}}}_{{\boldsymbol{c}},{\boldsymbol{t}}}^{\boldsymbol{^{\prime}}}{{\boldsymbol{\alpha}}}_{9}\\&+{{\boldsymbol{D}}{\boldsymbol{e}}{\boldsymbol{m}}{\boldsymbol{o}}}_{{\boldsymbol{c}},{\boldsymbol{t}}}^{\boldsymbol{^{\prime}}}{{\boldsymbol{\alpha}}}_{10}+{{\boldsymbol{D}}{\boldsymbol{i}}{\boldsymbol{s}}{\boldsymbol{t}}}_{{\boldsymbol{c}},{\boldsymbol{t}}}^{\boldsymbol{^{\prime}}}{{\boldsymbol{\alpha}}}_{11}\\&+{{\boldsymbol{G}}{\boldsymbol{P}}}_{{\boldsymbol{c}}}^{\boldsymbol{^{\prime}}}{{\boldsymbol{\alpha}}}_{12}+{{\boldsymbol{T}}}_{{\boldsymbol{t}}}^{\boldsymbol{^{\prime}}}{{\boldsymbol{\alpha}}}_{13}\big)\end{aligned}$$

where subscripts *c*, *t*, and *k* denote a given child, year (*t* = 2004…2010), and type of HCU (GP visits, specialist visits, diagnostic tests, or ER visits), respectively, and vector ${\boldsymbol{x}}$ comprises all the regressors, explained next. The dependent variables measure the annual number of visits or diagnostic tests of type *k* for child *c*. These outcomes are non-negative counts and exhibit a substantial mass at zero, particularly for specialist visits, diagnostic tests, and ER visits. Our specification therefore models the conditional mean of health care use. The estimated coefficients capture changes in expected utilization and thus reflect responses along both the extensive margin (any use) and the intensive margin (number of visits conditional on use). Parental socioeconomic status is measured in the following way: **MEduc** and **FEduc** are vectors of dummy variables for educational attainment of the mother and father, respectively; and *Inc* is a proxy for household labor income.^10^**MHCU** and **FHCU** are two vectors measuring utilization of different types of HCU (*k* = GP visits, Specialist visits, Diagnostic tests, or Emergency room visits) by the child’s mother and father.^11^ This makes it possible to investigate the existence of intergenerational transmission in HCU from mothers and fathers to their children, and separately by type of HCU.

We condition on other important variables that affect both children’s and parents’ HCU, the extension of which is, to our knowledge, unique in the literature. The most important reason for health service utilization is health status, which captures the individual need for health care (Álvarez & Vera-Hernández, 2013; Andersen, 1995).^12^ We measure child’s health status (**CH**) through the overall number of comorbidities in a given year plus a set of indicators of presence of severe diseases (CVDs such as hypertension, lipids and cholesterol problems, and cardio problems; bronchial asthma and chronic obstructive pulmonary disease; and the presence of any mental disorder). **PH** is a vector with parental health controls (number of parental comorbidities and indicators of whether any of the parents has a diagnosis of a mental health problem or has passed away). **CHB** and **PHB** include measures of children’s and parents’ health behaviors. These include indicators of whether the child is overweight, obese or has a missing information on BMI, as well as indicators of whether at least one parent is overweight, obese, or has a missing BMI information, smokes or is a heavy drinker. We include these parental health behavior variables as proxies for unobserved characteristics such as time preference of parents, as this is likely correlated with other included parental regressors (such as education and health) and many of their children’s outcomes, including HCU (Reinhold & Jürges, 2012).^13^ The vector **Demo** includes a set of demographic variables, namely, the age of the child, mother and father, and indicators of whether the child is female or immigrant. Our model also controls for the minimum distance to the child’s GP and assigned hospital (in km; vector **Dist**), in order to proxy for the cost of health care use in a system where health care is free at the point of use (Álvarez & Vera-Hernández, 2013).^14^ Finally, vectors **T** and **GP** include, respectively, calendar year dummies and specific GP fixed effects. The latter have been used as a measure of physicians’ practice styles (e.g. Ahammer & Schober, 2020) and will capture ‘supplier-induced demand’, that is, GPs influencing patients’ demand for care.

We estimate Eq. (1) using the Poisson quasi-maximum likelihood estimator, which is robust to overdispersion in the dependent variable, as well as Poisson quasi-conditional maximum likelihood to estimate a specification that additionally allows the inclusion of high-dimensional fixed effects such as child FEs (Wooldridge, 1999). This specification accounts for any remaining time-invariant unobserved family factors, including certain parental attitudes and preferences, which may drive correlation between child and parents’ health care use. These estimates are informative of the extent to which there are common patterns in the reaction of parental and child health care to common (or correlated) shocks. For the latter, we use the estimation procedure proposed by Correia et al. (2020). All analyses were performed for the full sample of all children, as well as separately for the sub-samples of daughters and sons. In the next section, we discuss the results from our preferred model specification, Eq. (1). Nevertheless, in the Appendix we also show results of specifications excluding some sets of parental and child regressors (health status and health behaviors). We do so, first, to facilitate comparison with previous studies and, secondly, because those variables may be “bad controls”, as they may be themselves intermediate outcomes of parental variables of interest such as education and income (Propper et al., 2007; Reinhold & Jürges, 2012).

Given the number of estimated coefficients across outcomes and model specifications, we address concerns regarding multiple hypothesis testing by applying the Benjamini–Hochberg false discovery rate (FDR) procedure (Benjamini & Hochberg, 1995) to the p-values associated with the parental health care utilization variables (father's and mother's GP visits, specialist visits, and emergency visits). The multiple-testing correction is applied jointly to all parental health care utilization coefficients, which represent the parameters of primary interest in the analysis, across all outcomes and model specifications. FDR-adjusted q-values are reported in Appendix Tables A10 and A11.

## Results

**Fig. 1:**
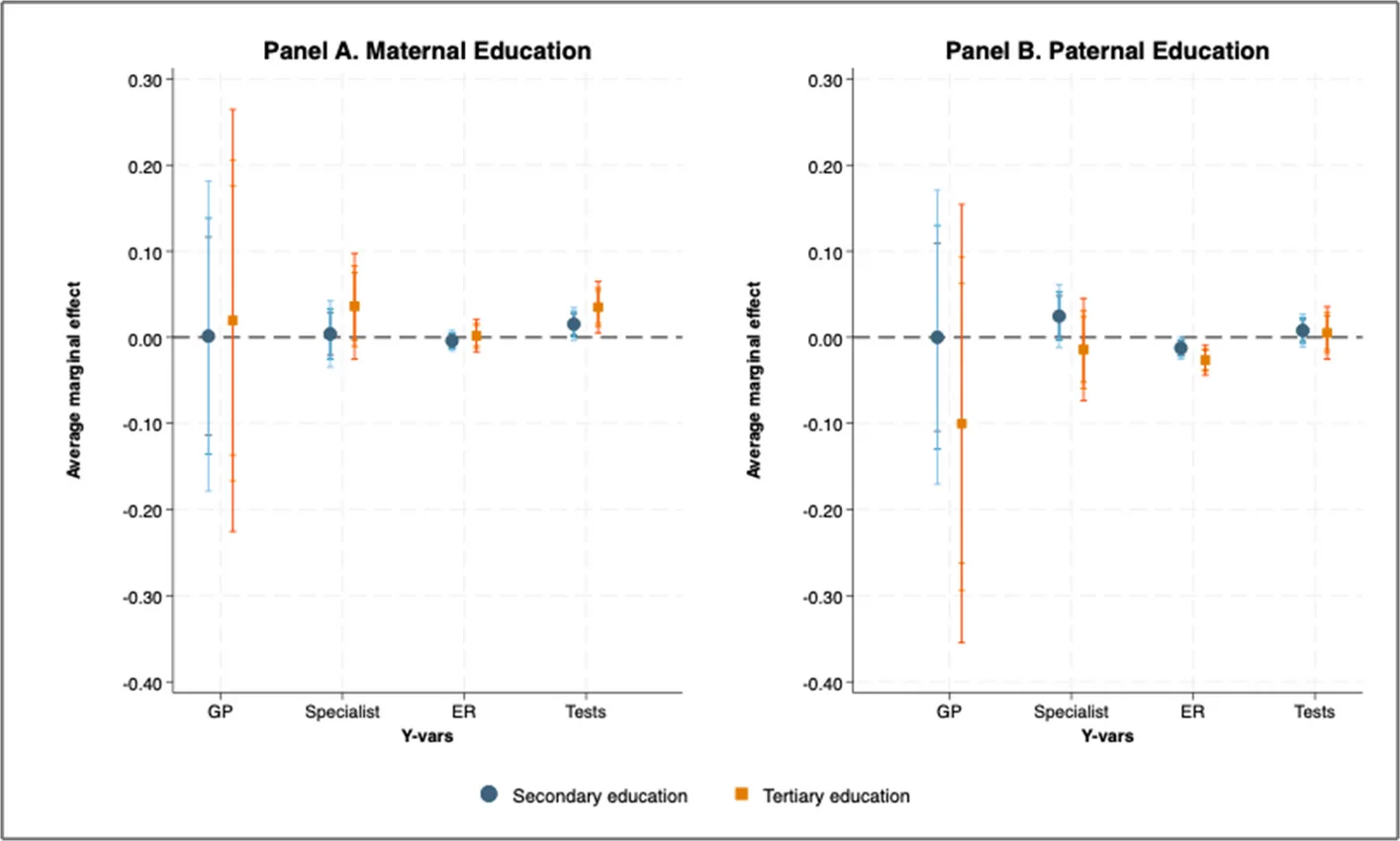
Marginal effects of maternal and parental education levels on their children’s type of health care use. Notes: Average marginal effects from Poisson models with 90, 95 and 99% confidence intervals based on robust standard errors (full results shown in Appendix Tables A1- A4, Model 4)

**Fig. 2:**
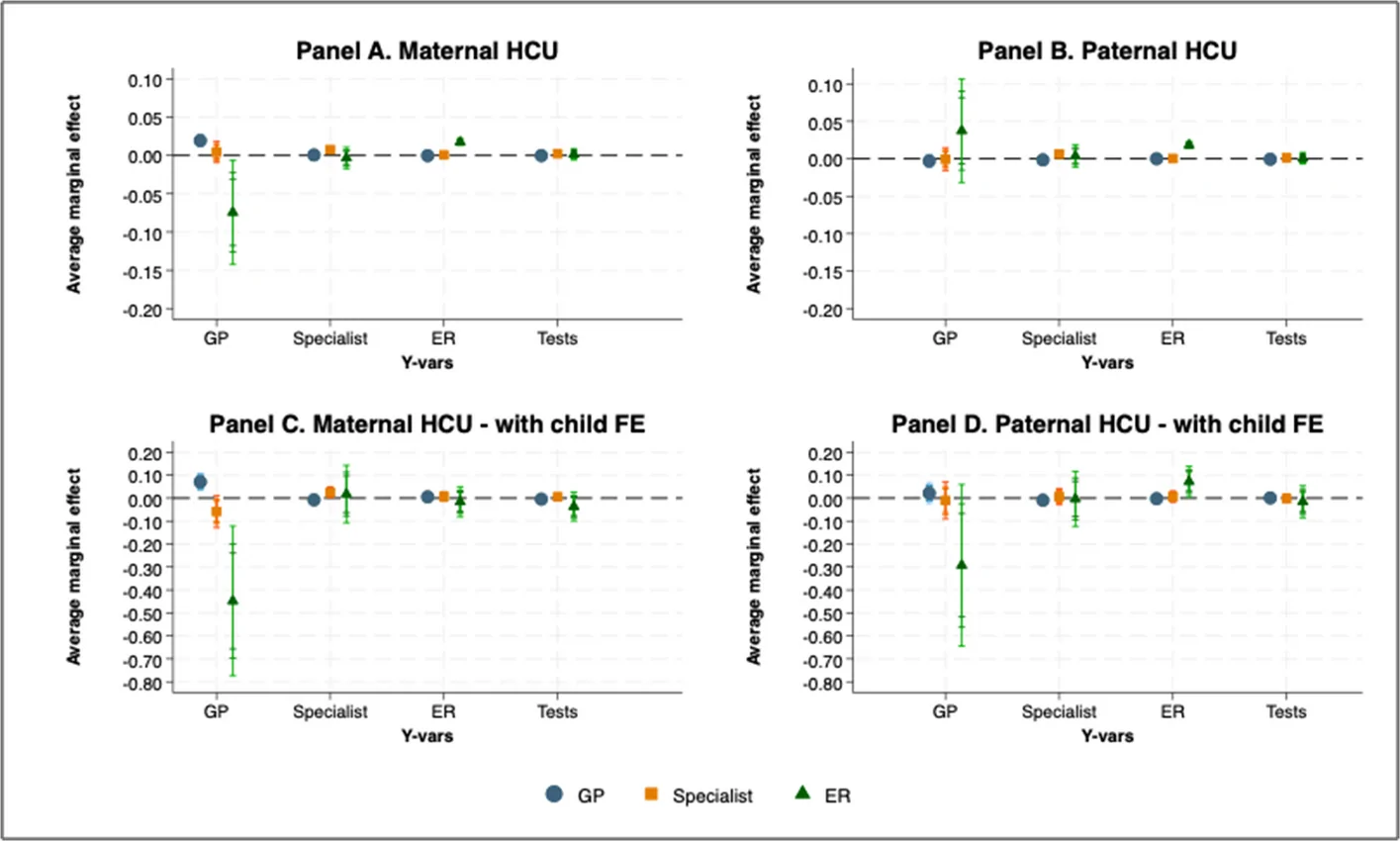
Marginal effects of parental health care use on their children’s type of health care use (HCU) – with and without child fixed effects (FE). Notes: Average marginal effects from Poisson models (Panels A and B) and from Poisson models with child fixed effects (Panels C and D) with 90, 95 and 99% confidence intervals based on robust standard errors (full results shown, respectively, in Appendix Tables A1-A4 and A5, A6, Model 4)

**Fig. 3:**
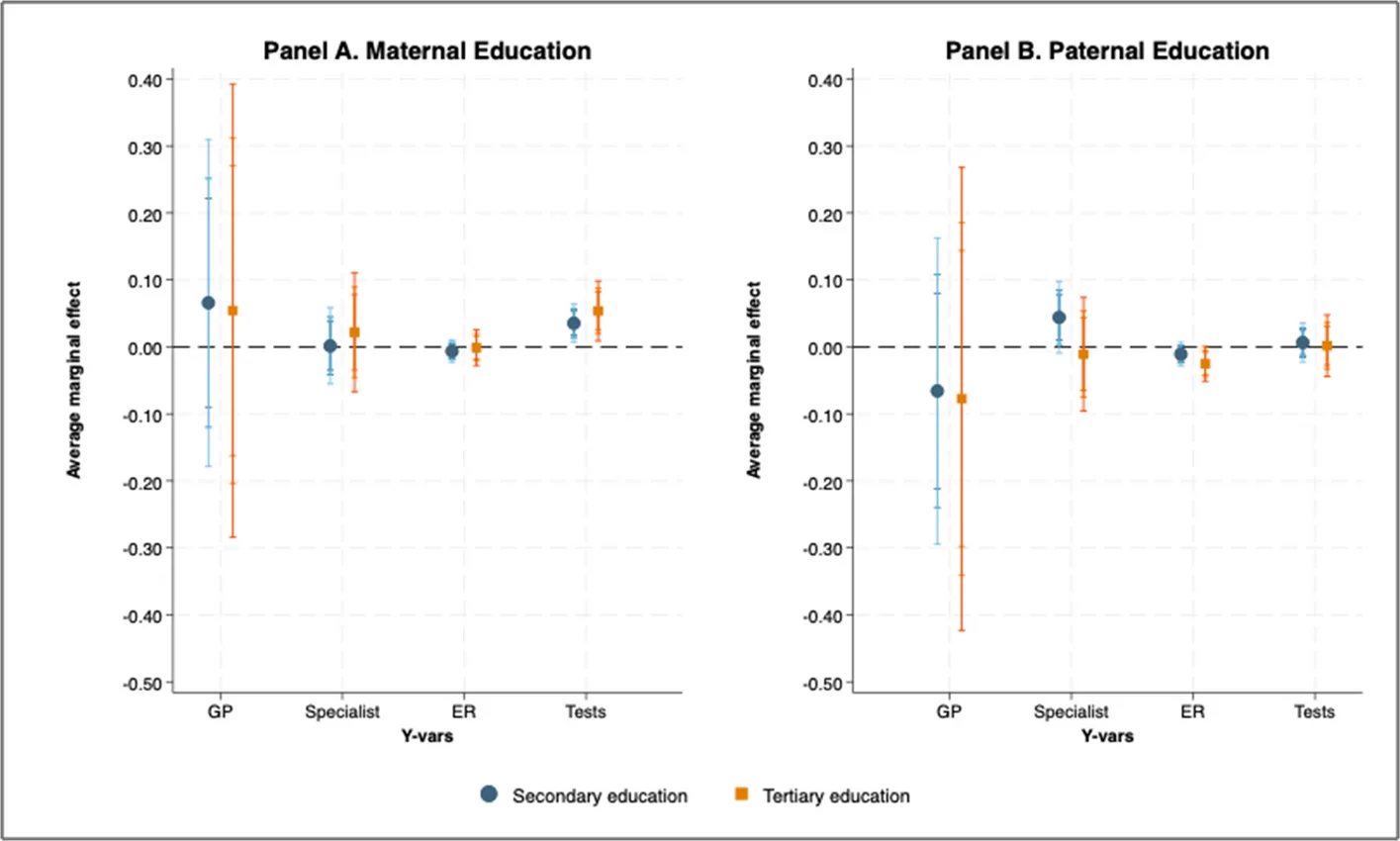
Marginal effects of maternal and parental education levels on their daughters’ type of health care use. Notes: Average marginal effects from Poisson models with 90, 95 and 99% confidence intervals based on robust standard errors (full results shown in Appendix Tables A7 and A8, Model 4)

**Fig. 4:**
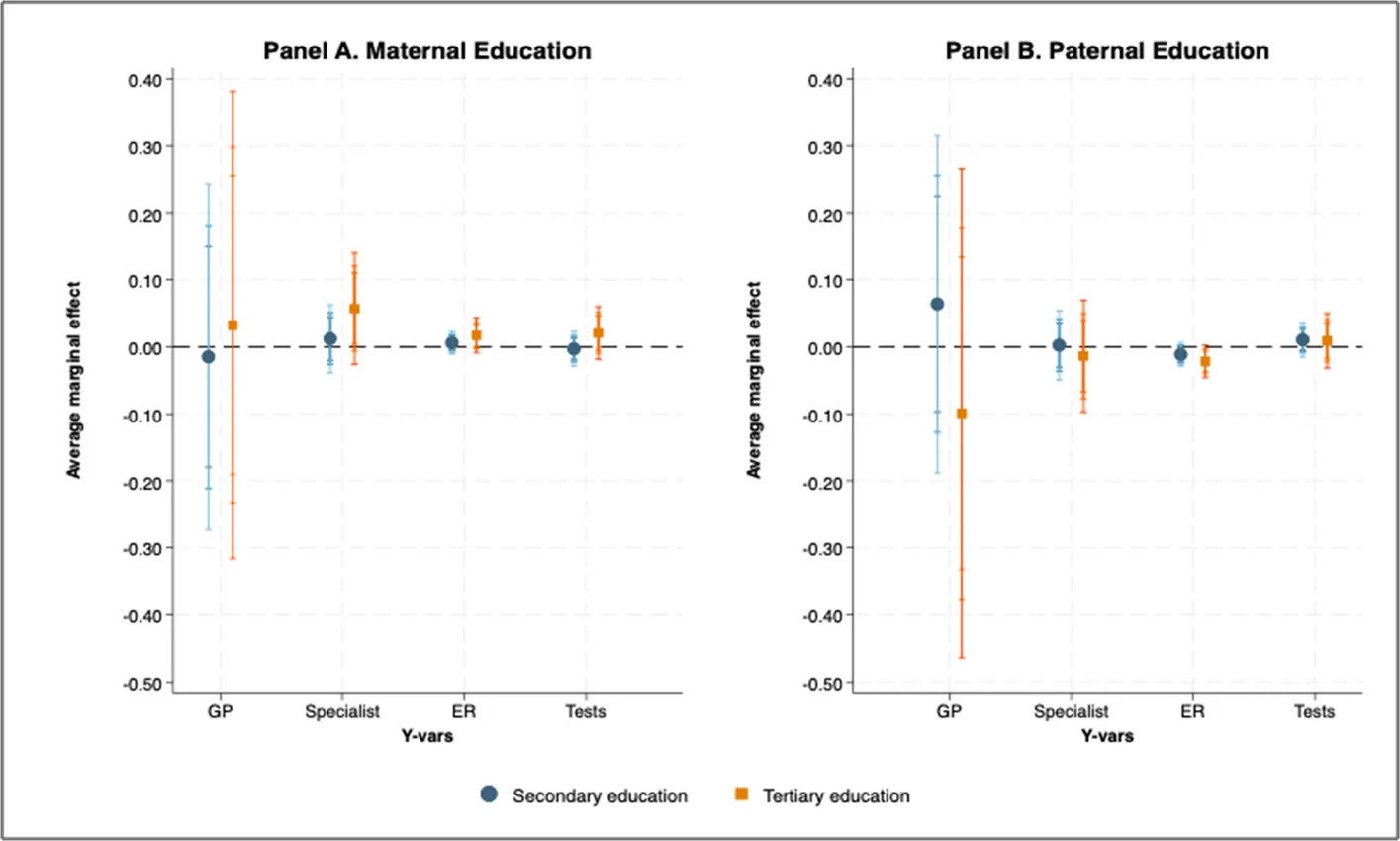
Marginal effects of maternal and parental education levels on their sons’ type of health care use. Notes: Average marginal effects from Poisson models with 90, 95 and 99% confidence intervals based on robust standard errors (full results shown in Appendix Tables A7 and A8, Model 4)

**Fig. 5:**
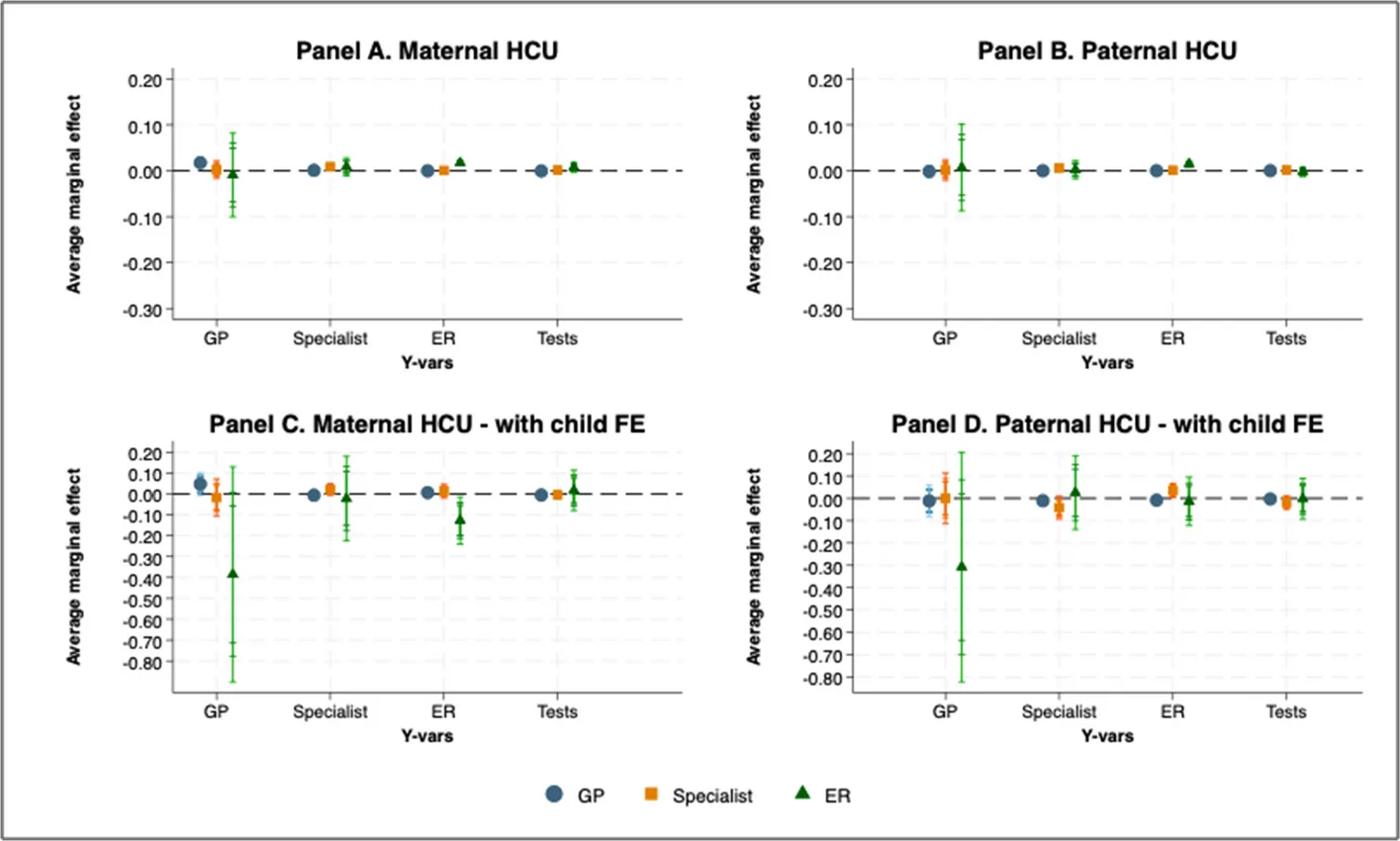
Marginal effects of parental health care use on their daughters’ type of health care use (HCU) – with and without child fixed effects (FE). Notes: Average marginal effects from Poisson models (Panels A and B) and from Poisson models with child fixed effects (Panels C and D) with 90, 95 and 99% confidence intervals based on robust standard errors (full results shown, respectively, in Appendix Tables A7, A8 and A9, Model 4)

**Fig. 6:**
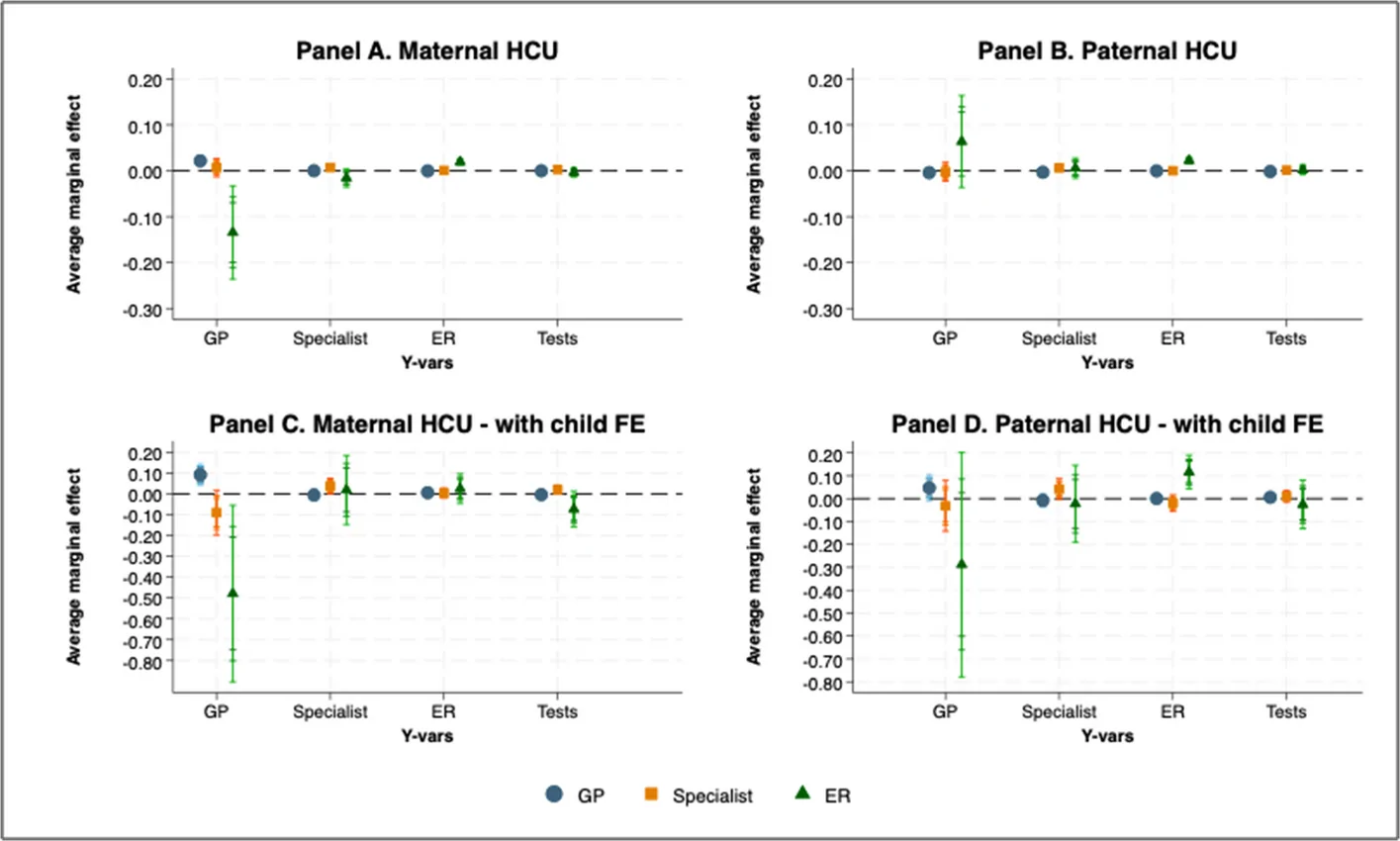
Marginal effects of parental health care use on their sons’ type of health care use (HCU) – with and without child fixed effects (FE). Notes: Average marginal effects from Poisson models (Panels A and B) and from Poisson models with child fixed effects (Panels C and D) with 90, 95 and 99% confidence intervals based on robust standard errors (full results shown, respectively, in Appendix Tables A7, A8 and A9, Model 4)

This section presents and discusses our main findings. Figures 1, 2, 3, 4, 5 and 6 show the main estimates of interest according to our preferred specification—Eq. (1). These consist of average marginal effects of maternal and paternal education and different types of HCU on the expected HCU of their children, conditional on child and parental health and behaviors, child and parental demographic variables, calendar year indicators and supply factors (distance to the child’s GP and reference hospital, and GP fixed effects). As discussed in the previous section, we estimate Poisson models with and without child FEs. We first present the results for the full sample of all children (Figs. 1 and 2), and then separately for daughters (Figs. 3 and 5) and sons (Figs. 4 and 6).

Appendix Tables A1-A9 show full results of that preferred specification, as well as specifications with alternative sets of control variables. The baseline specification (Model 1) estimates associations with parental SES and HCU variables conditional on a set of basic controls (child and parental demographics, calendar year indicators, and supply factors). Model 2 includes additionally health and lifestyle factors of children and Model 3 also those of parents. As explained above, the preferred specification includes the full set of regressors (Eq. (1), Model 4).

### Association of children’s health care use with parental socioeconomic status

Figure 1 shows average marginal effects of mothers’ and fathers’ educational attainment on their children’s HCU using our preferred specification. We find that, conditional on all other observed child and parent covariates, children with more educated mothers get on average more diagnostic tests than those with less educated mothers (Panel A) and that children with more educated fathers have less ER visits than those with less educated fathers (Panel B). These differences between low and high educated mothers and fathers are substantial, amounting correspondingly to 0.036 (i.e., 12%) more diagnostic tests and 0.023 (20%) less ER visits. Moreover, these estimates and their statistical significance are not very sensitive to excluding parental and child health and lifestyle factors (Models 1–3, Table A1). For other types of HCU, we find no evidence of an association with mothers’/fathers’ educational level. In particular, we find no evidence of an association with specialist visits, and with GP visits after controlling for child health and lifestyle factors.

We also find a negative correlation between parental labor income and GP visits, specialist visits and diagnostic tests, although the marginal effects are modest (smaller than a 0.3% reduction in visits for each additional 1000€ or 3% increase in parental labor income; Table 1). While this may appear at odds with the broader literature on socioeconomic gradients in health care use, the pattern is consistent with substitution toward private care in Spain’s mixed public–private system. Notably, we find no income gradient in children’s emergency room visits (Table 1), a type of care predominantly delivered through the public hospital network and along which substitution to private providers is more limited. By contrast, the negative association is strongest for GP visits, precisely the margin where households with supplementary private insurance are more likely to substitute away from public providers. This interpretation aligns with evidence from Spain showing that individuals with dual coverage rely less on public primary care and display different utilization patterns across GP and specialist services (Cantarero-Prieto et al., 2017; González Álvarez & Clavero Barranquero, 2009). Because our administrative data capture only public utilization, greater reliance on private GP services among higher-income families would translate into lower observed public use without implying reduced overall access to care.

### Is there an intergenerational transmission in health care use?

One of the key aims of this paper is to investigate the association between the use of specific types of health care services for mothers and fathers with those of their children, particularly, after including a broad set of controls related to children’s (and parents’) health needs, parents’ SES, and supply-side factors as captured by GP FEs. Figure 2 depicts these results (without child-fixed effects) in Panels A and B.

For specialist and ER visits, we find evidence of a mother/father-child association, but the magnitudes are relatively small. One additional visit to a specialist by mothers and fathers (about a 30% increase in these visits for both) is associated with an average increase of 0.008 visits (2%) and 0.006 visits (1%) to a specialist by their children, respectively, controlling for all other child and parent covariates. The association for ER visits are slightly larger, namely, a one-unit increase in ER visits among mothers and fathers (around a 145% increase) is associated with about 0.018 increase in their children’s ER visits (15%). These estimates and their statistical significance remain unchanged when we exclude parental and child health and lifestyle factors (Models 1–3, Table A2).

Interestingly, only mothers’ HCU seems to play a role in explaining children’s GP visits (note that children in the SNHS have their own GP, which is a different one from that of their parents). We find a positive mother–child associations for GP visits but a negative one between mothers’ ER visits and their children’s GP visits, conditional on all other child and parent covariates. The coefficients, however, are small. For instance, a one-unit (i.e., 144%) increase in mothers’ ER visits is associated just with a 0.074 (1%) decline in children’s GP visits. Again, excluding subsets of control variables changes little these estimates or their statistical significance (Models 1–3, Table A2).

To what extent are these mother/father-child associations driven by (time-constant) unobserved factors such as parents’ preferences for (certain types of) health care? To address this question, we estimate Poisson models with child FEs (Fig. 2, Panels C and D). This somewhat strengthens the pattern of association between children’s GP visits and their mothers HCU. A one-unit (or 144%) increase in mothers’ ER visits is associated with a 0.45 (7%) decline in child GP visits. We now observe a significant negative association, though smaller in absolute terms (0.29, 4%), between fathers’ ER visits and their children’s GP visits. For specialist and ER visits, we now find, respectively, statistically significant mother–child and father-child associations only (instead of mother/father-child association for both types of care). These estimates are nevertheless 3–4 times larger than in the models without child FEs. One additional visit to a specialist by mothers (about a 30% increase) is associated with a 0.026 (6%) increase in children’s specialist visits. For ER, a one-unit (or 145%) increase in fathers’ visits is associated with about 0.072 (62%) increase in their children’s expected ER visits.

### Is the intergenerational transmission of health care use larger within same-sex parent–child dyads?

This section investigates if the earlier documented intergenerational transmission of health care use is larger within same-sex parent–child dyads. We show results of all specifications estimated separately by child sex for daughters and sons in Appendix Tables A7-A9 and of the preferred specification (Model 4) in Figs. 3 and 5 for daughters and in Figs. 4 and 6 for sons.

Overall, the associations between mothers’/fathers’ education and their children’s health care use remain small when performing separate analyses by child sex, but some additional insights are gained (Figs. 3 and 4). For instance, we show that the result that children with more educated mothers get more diagnostic tests than those with less educated mothers is driven by daughters. On the other hand, both boys and girls with more educated fathers have less ER visits than those with less educated fathers. Additionally, a negative correlation with parental labor income is observed with both daughters’ and sons’ health care use (Table A7).

Only mothers’ health care use (specifically, GP and ER visits) appears to play a role in explaining their children’s GP visits. The associations between mothers’ and children’s GP visits are symmetric by child sex; on the other hand, the negative correlation between mothers’ ER visits and their children’s GP visits is driven by sons only (Panels A in Figs. 5 and 6). These findings are robust to the inclusion of child FEs; in fact, the patterns become somewhat stronger (Panels C in Figs. 5 and 6). For fathers, we find an association between theirs and their daughters’ ER visits. However, this association is fully captured by time-constant unobserved factors (Panels B and D in Fig. 5). On the other hand, the association between paternal health care use and that of their sons becomes stronger after controlling for time-constant unobserved factors (Panels B and D in Fig. 6). In sum, we find evidence of intergenerational associations within types of health care use for GP visits, specialists and, especially, for ER visits, which for fathers are restricted to same-sex parent–child dyads when child FEs are controlled for.^15^

## Conclusion

This paper uses rich longitudinal administrative medical records to examine the separate roles of mothers and fathers in children’s different types of health care use. We further distinguish according to child sex, which allows us to investigate whether the intergenerational transmission of health care use is larger within same-sex parent–child dyads. In addition, we investigate if these associations are driven by time-constant unobserved factors, such as parental preferences regarding health care. All our findings are robust to inclusion of an extensive set of control variables related to children’s and parental health needs and health behaviors. This study has some limitations. Most notably, while our data are rich in several important respects, they cover only one (albeit) large municipality in Spain, Badalona. Whereas this municipality is fairly representative of the Spanish population in terms of socioeconomic status, this still potentially reduces generalizability. Nevertheless, our findings, we believe, expand the previous knowledge on socioeconomic inequalities in children’s health care use in several respects.

Overall, we find small associations between health care use of children and socioeconomic status of their parents. There is limited or no evidence of a correlation with parents’ education and mostly a negative correlation with parental labor income. One exception to this is the strong positive association between children’s diagnostic tests and mothers’ educational attainment (which appears to be driven by daughters). Taken together, and given that our data cover public health providers, these results are consistent with two mechanisms: greater access to private health care by children of parents with higher socioeconomic status; and a higher preference of higher educated mothers for more extensive health care for their children (and, more specifically, for their daughters), compared to their lower educated counterparts. Relatedly Bosque‐Mercader et al. (2023), also using data from Catalonia, find evidence of shorter waiting times for various publicly funded hospital surgeries among patients with a higher SES. These patients, they argue, “may have a better understanding and be more familiar with the administrative processes to access specialist services, put pressure to the provider through frequent phone calls or rely on informal channels (e.g., knowing someone working at the hospital)” (p. 1182).

Regarding parental health care use, we show associations with their children’s health care use which tend to occur within the same type of health care. Moreover, for some types of care (GP and specialist visits), we find evidence of intergenerational transmission in health care use within the same-sex parent–child dyads. Nevertheless, in general, mothers’ health care use is more strongly associated with that of their children. For mothers, we also uncovered a negative association between their ER visits (which are typically unplanned) and their children’s GP visits (which are typically planned), a pattern driven by sons only. One possible interpretation is substitution between planned and unplanned care within the household. However, given the number of estimated associations and the potential for multiple mechanisms to operate simultaneously, this pattern should be interpreted with caution and may reflect the interaction of several mechanisms rather than a single behavioral channel.

We find that these associations mostly remain, and even sometimes strengthen, after controlling for unobserved time-invariant factors, such as parental preferences or background. Our models include a large range of parental and child health variables. This suggests that common family shocks – over and above changes in parental or children health—may also play an important role in shaping health care use patterns. It is not possible to shed light on the nature of such mechanisms in this paper. These may include socioeconomic events such as parental job loss or changes in risk attitudes, driven for example by health shocks to friends or family. Investigation of the role of such possible mechanisms remains a topic for future research.

Our findings are informative for policies aimed at reducing health inequalities. For instance, our result of intergenerational transmission in health care use from mothers and fathers to their daughters and sons has potentially important policy implications, as patterns of health care seeking are established early in life, which the literature shows have long-lasting effects over people’s life course.

## Supplementary Information

Below is the link to the electronic supplementary material.

Supplementary file1 (DOCX 79 KB)

## Footnotes

1 Most medical studies rely on the so-called “behavioral model of health services use” (Aday & Andersen 1981; Andersen 1995) when investigating the determinants of children’s health care use. This model identifies three primary classes of barriers to access and intensity of use of health care services: enabling factors (those financial and effort factors that influence the accessibility of care such as income, the availability of insurance coverage, and proximity to health facilities), need factors (children’s health), and predisposing factors (individual characteristics that exist prior to seeking care and include children’s age, sex, and race, family size and several parental characteristics, often limited to mothers, and related to their educational level, employment status, social support, health status and own health care use).

2 A related literature from low- and middle-income countries, largely based on Demographic and Health Surveys (DHS), documents pronounced socioeconomic inequalities in access to and coverage of maternal and child health services, often stratified by household wealth and maternal education (i.e., Barros et al., 2012; Victora et al., 2003, 2012). While this evidence provides important descriptive insights into socioeconomic gradients in child-related health services, it is largely cross-sectional, relies on self-reported measures, and does not examine health care utilization conditional on health needs or intergenerational transmission mechanisms. As a result, it offers limited scope to control for child and parental health status or unobserved family characteristics, underscoring the value of studies using richer longitudinal administrative data.

3 Controlling for health status (i.e., need for health care) has been shown to be crucial for conclusions regarding socioeconomic-related inequality in health care use of adults. For example, Van Doorslaer et al. (2004) find mostly pro-poor inequality in the number of GP visits in 12 European countries, which becomes smaller or disappears after standardizing for health status. A similar pattern occurs for specialists; for 10 of the countries studied in that paper there is evidence of pro-rich inequality when standardizing for health status; for several countries, this emerges only after standardization; for the remaining countries that exhibit pro-rich inequality even before standardization, this is underestimated.

4 These studies tend to obtain health care data from parental reports which are more prone to measurement error and reporting bias than similar data from administrative medical records (see e.g. Bound, Brown & Mathiowetz, 2001) used here and considered a gold standard in terms of data accuracy. Exceptions are Minkovitz et al. (2005) who use also medical records for preventive care visits but parent interviews for hospitalizations and emergency department visits, and Janicke et al. (2001) and Riley et al. (1993) who obtain the number of health care visits by children enrolled in a health maintenance organization from the computerized encounter system of the corresponding health care plan. Saxena et al. (1999) have a large sample of 106102 children with validated consultation data that were prospectively collected over 1 year by volunteer GPs and nurses. Olsen et al. (2021) and Petrou and Kupek (2005) use hospital records to measure hospital inpatient service utilization (number of admissions and days) and average annual costs for a large sample of children in two counties of southern England and for a full cohort of children in Norway, respectively.

5 For instance, Minkovitz et al. (2005) find evidence of higher attrition among mothers with depressive symptoms and Berra et al. (2009) find a lower response rate among low-educated parents.

6 Other related studies have investigated the impacts of income (Reinhold and Jürges 2012) and eligibility to public health insurance (De La Mata 2012) on children’s preventive HCU in Germany and in the U.S., respectively. Another recent study has assessed the effect of cost-sharing on different types of children’s HCU in Sweden (Nilsson and Paul 2018). Two of these exploit discontinuities in income eligibility rules (De La Mata 2012) and age thresholds below which copayments are not charged along with two policy changes in cost-sharing for children (Nilsson and Paul 2018). All of them include only limited information on parents (they, for instance, omit parental HCU) and, more generally, do not study the potential roles that fathers and mothers may play in explaining their children’s HCU and do not explore differences by child sex.

7 The six primary care centres included in our data are Apenins-Montigalà, Morera-Pomar, Montgat-Tiana, Nova Lloreda, Progrés-Raval and Marti i Julià), and the two reference hospitals are Hospital Municipal de Badalona and Hospital Universitari Germans Trias i Pujol. Two recent studies using similar data to ours have studied lifestyle behaviors and health care use in adults (Gaggero et al., 2022; Gil et al., 2019).

8 We use clinical or measured information on weight and height to compute individuals’ body mass index (BMI). Self-reporting error in observed BMI is therefore not an issue here. However, while some individuals get several measurements (of height and weight) during our sample period, others are never measured. We control for missing information on BMI in our empirical models.

9 Health care use is measured annually. Although this may reflect short-term shocks, our empirical models in Sect. "Econometric model" include detailed health controls, year fixed effects, and—in some specifications—child fixed effects, which mitigate concerns about transitory correlated shocks. Any additional noise from annual aggregation would tend to attenuate estimated associations.

10 We are interested mainly in the associations with parental education keeping their income fixed. This is relevant as, especially education level, is likely to capture both ability to pay and preferences regarding health care. Our results are robust to including parental schooling years or categories of educational attainment (available upon request). Given that our parents are on average in their 40 s (see Table 1), we treat education as a predetermined variable throughout the analysis. We proxy household labor income using information on the parent’s occupation available in the Census of 2001 and the profiles on average earnings by occupation in 2002 for Catalonia provided by Idescat.

11 We could unfortunately not retrieve information on diagnostic tests for the parents.

12 Nevertheless, economists typically exclude health need variables from (children’s) HCU equations as these are potential outcomes of other included regressors such as (parental) income and education (cf. Currie et al., 2008; Reinhold & Jürges 2012).

13 Dohmen et al. (2012) find evidence of intergenerational transmission of risk preferences.

14 Health care is free at the point of access in Spain but an opportunity cost for HCU still exists. It is unclear, however, whether high- and low-SES parents are affected differently. While one may argue that high-wage (or high-SES) parents have higher opportunity costs and might thus be less likely to have their children examined, low-income (or low-SES) parents may have higher opportunity costs despite lower wages because of differential penalties for missing work in lower skilled jobs compared with a professional position (Reinhold & Jürges 2012).

15 After applying the Benjamini–Hochberg false discovery rate (FDR) correction, the strongest and most robust associations correspond to the intergenerational transmission of general practitioner utilization. Parental GP visits—particularly maternal visits—remain highly significant across specifications (with q-values well below 1%), indicating a stable association between parental and child use of primary care services. Several additional associations survive the FDR correction at the 10% level, including maternal specialist visits for child specialist use and parental emergency care utilization in some specifications. In contrast, associations involving diagnostic tests and emergency care are more sensitive to model specification and generally remain significant only at more permissive FDR thresholds, suggesting weaker or less stable transmission patterns once multiple testing is taken into account (see Appendix Tables A10 and A11).
