# Supplementary material for: The role of mothers and fathers in children’s health care use
Source: Int J Health Econ Manag. 2026 Jun 29;26(3):13. doi: 10.1007/s10754-026-09417-x (PMC13314905; doi:10.1007/s10754-026-09417-x)
Supplement: Supplementary file 1 — Supplementary file1 (DOCX 79 KB) [file 10754_2026_9417_MOESM1_ESM.docx]

**Online Appendix**

**Table A1. Average marginal effects of parental education and household labor income on their children’s health care use**

|  | Child GP visits | | | | Child specialist visits | | | | Child diagnostic tests | | | | Child ER visits | | | |
| --- | --- | --- | --- | --- | --- | --- | --- | --- | --- | --- | --- | --- | --- | --- | --- | --- |
|  | (M1) | (M2) | (M3) | (M4) | (M1) | (M2) | (M3) | (M4) | (M1) | (M2) | (M3) | (M4) | (M1) | (M2) | (M3) | (M4) |
| Father secondary (0-1) | 0.242 | 0.043 | 0.093 | -0.002 | 0.031 | 0.024 | 0.024 | 0.022 | 0.016 | 0.010 | 0.009 | 0.007 | -0.014 | -0.014 | -0.012 | -0.012 |
|  | (0.105) | (0.066) | (0.103) | (0.067) | (0.015) | (0.014) | (0.016) | (0.014) | (0.008) | (0.007) | (0.008) | (0.007) | (0.005) | (0.005) | (0.005) | (0.005) |
| Father tertiary (0-1) | -0.345 | -0.094 | -0.304 | -0.105 | -0.039 | -0.013 | -0.037 | -0.014 | -0.012 | 0.006 | -0.010 | 0.005 | -0.031 | -0.026 | -0.028 | -0.023 |
|  | (0.164) | (0.098) | (0.157) | (0.099) | (0.023) | (0.023) | (0.024) | (0.023) | (0.013) | (0.012) | (0.013) | (0.012) | (0.007) | (0.007) | (0.007) | (0.007) |
| Mother secondary (0-1) | 0.383 | 0.027 | 0.323 | 0.016 | 0.003 | 0.000 | 0.009 | 0.003 | 0.021 | 0.015 | 0.023 | 0.015 | -0.000 | -0.000 | 0.001 | 0.000 |
|  | (0.109) | (0.070) | (0.106) | (0.070) | (0.016) | (0.015) | (0.016) | (0.015) | (0.008) | (0.007) | (0.008) | (0.007) | (0.005) | (0.005) | (0.005) | (0.005) |
| Mother tertiary (0-1) | 0.025 | 0.012 | 0.147 | 0.037 | -0.005 | 0.029 | 0.017 | 0.037 | 0.013 | 0.032 | 0.026 | 0.036 | -0.000 | 0.005 | 0.005 | 0.007 |
|  | (0.153) | (0.095) | (0.147) | (0.095) | (0.024) | (0.023) | (0.025) | (0.024) | (0.013) | (0.012) | (0.013) | (0.012) | (0.007) | (0.007) | (0.007) | (0.007) |
| Inferred parental salaries | -0.015 | -0.005 | -0.014 | -0.005 | -0.002 | -0.001 | -0.002 | -0.001 | -0.001 | -0.000 | -0.001 | -0.000 | -0.000 | -0.000 | -0.000 | -0.000 |
|  | (0.002) | (0.001) | (0.002) | (0.001) | (0.000) | (0.000) | (0.000) | (0.000) | (0.000) | (0.000) | (0.000) | (0.000) | (0.000) | (0.000) | (0.000) | (0.000) |
| Basic controls and GP fixed effects | Yes | Yes | Yes | Yes | Yes | Yes | Yes | Yes | Yes | Yes | Yes | Yes | Yes | Yes | Yes | Yes |
| Parental health care use | Yes | Yes | Yes | Yes | Yes | Yes | Yes | Yes | Yes | Yes | Yes | Yes | Yes | Yes | Yes | Yes |
| Child health and lifestyle | - | Yes | - | Yes | - | Yes | - | Yes | - | Yes | - | Yes | - | Yes | - | Yes |
| Parental health and lifestyle | - | - | Yes | Yes | - | - | Yes | Yes | - | - | Yes | Yes | - | - | Yes | Yes |
| Log. pseudolikelihood | -434538 | -298753 | -424914 | -298092 | -85823 | -79138 | -85251 | -79037 | -64894 | -58143 | -64362 | -58088 | -29978 | -29146 | -29911 | -29092 |
| Observations | 93365 | 93365 | 93365 | 93365 | 93365 | 93365 | 93365 | 93365 | 93365 | 93365 | 93365 | 93365 | 93365 | 93365 | 93365 | 93365 |

Notes: Marginal effects from four Poisson model specifications. Specifications 1-4 are designated as M1-M4. Robust standard errors in parentheses. Each set of included variables is described in Table 1, except that child age is defined via single year dummies for ages 0-1 and biannual year dummies for ages 2-18, rather than a continuous age variable. All models include also fixed effects (FE) for the 80 GP providers for this population.

**Table A2. Average marginal effects of parental health care use on their children’s type of health care use**

|  | Child GP visits | | | | Child specialist visits | | | | Child diagnostic tests | | | | Child ER visits | | | |
| --- | --- | --- | --- | --- | --- | --- | --- | --- | --- | --- | --- | --- | --- | --- | --- | --- |
|  | (M1) | (M2) | (M3) | (M4) | (M1) | (M2) | (M3) | (M4) | (M1) | (M2) | (M3) | (M4) | (M1) | (M2) | (M3) | (M4) |
| Fathers' visits to GP | 0.041 | 0.010 | -0.008 | -0.003 | 0.002 | -0.000 | -0.002 | -0.002 | 0.002 | 0.000 | -0.001 | -0.001 | 0.000 | -0.000 | 0.000 | 0.000 |
|  | (0.006) | (0.003) | (0.005) | (0.003) | (0.001) | (0.001) | (0.001) | (0.001) | (0.000) | (0.000) | (0.000) | (0.000) | (0.000) | (0.000) | (0.000) | (0.000) |
| Mothers' visits to GP | 0.089 | 0.032 | 0.047 | 0.019 | 0.005 | 0.001 | 0.003 | 0.001 | 0.004 | 0.001 | 0.001 | -0.000 | 0.000 | -0.000 | 0.000 | -0.000 |
|  | (0.007) | (0.003) | (0.005) | (0.003) | (0.001) | (0.001) | (0.001) | (0.001) | (0.000) | (0.000) | (0.000) | (0.000) | (0.000) | (0.000) | (0.000) | (0.000) |
| Fathers' visits to specialist | 0.041 | 0.008 | 0.009 | -0.000 | 0.008 | 0.006 | 0.007 | 0.006 | 0.003 | 0.002 | 0.002 | 0.001 | 0.000 | -0.000 | 0.001 | 0.000 |
|  | (0.009) | (0.006) | (0.009) | (0.006) | (0.001) | (0.001) | (0.001) | (0.001) | (0.001) | (0.001) | (0.001) | (0.001) | (0.000) | (0.000) | (0.000) | (0.000) |
| Mothers' visits to specialist | 0.060 | 0.006 | 0.040 | 0.004 | 0.010 | 0.008 | 0.009 | 0.008 | 0.005 | 0.003 | 0.004 | 0.002 | 0.001 | 0.001 | 0.001 | 0.001 |
|  | (0.009) | (0.005) | (0.008) | (0.005) | (0.001) | (0.001) | (0.001) | (0.001) | (0.001) | (0.001) | (0.001) | (0.001) | (0.000) | (0.000) | (0.000) | (0.000) |
| Fathers' ER visits | 0.075 | 0.060 | -0.003 | 0.037 | 0.001 | 0.002 | 0.002 | 0.004 | 0.001 | 0.001 | -0.001 | 0.001 | 0.017 | 0.017 | 0.018 | 0.018 |
|  | (0.040) | (0.026) | (0.038) | (0.027) | (0.006) | (0.006) | (0.006) | (0.006) | (0.003) | (0.003) | (0.003) | (0.003) | (0.002) | (0.002) | (0.002) | (0.002) |
| Mothers' ER visits | -0.036 | -0.066 | -0.111 | -0.074 | 0.002 | -0.002 | -0.002 | -0.003 | 0.005 | 0.002 | 0.001 | 0.001 | 0.018 | 0.018 | 0.018 | 0.018 |
|  | (0.043) | (0.026) | (0.038) | (0.026) | (0.006) | (0.006) | (0.006) | (0.005) | (0.003) | (0.003) | (0.003) | (0.003) | (0.002) | (0.002) | (0.002) | (0.002) |
| Basic controls and GP fixed effects | Yes | Yes | Yes | Yes | Yes | Yes | Yes | Yes | Yes | Yes | Yes | Yes | Yes | Yes | Yes | Yes |
| Parental education and wages | Yes | Yes | Yes | Yes | Yes | Yes | Yes | Yes | Yes | Yes | Yes | Yes | Yes | Yes | Yes | Yes |
| Child health and lifestyle | - | Yes | - | Yes | - | Yes | - | Yes | - | Yes | - | Yes | - | Yes | - | Yes |
| Parental health and lifestyle | - | - | Yes | Yes | - | - | Yes | Yes | - | - | Yes | Yes | - | - | Yes | Yes |
| Log. Pseudolikelihood | -434538 | -298753 | -424914 | -298092 | -85823 | -79138 | -85251 | -79037 | -64894 | -58143 | -64362 | -58088 | -29978 | -29146 | -29911 | -29092 |
| Observations | 93365 | 93365 | 93365 | 93365 | 93365 | 93365 | 93365 | 93365 | 93365 | 93365 | 93365 | 93365 | 93365 | 93365 | 93365 | 93365 |

Notes: As Table A1.

**Table A3. Average marginal effects of parental lifestyles on their children’s type of health care use**

|  | Child GP visits |  | Child specialist visits | | Child diagnostic tests | | Child ER visits |  |
| --- | --- | --- | --- | --- | --- | --- | --- | --- |
|  | (M3) | (M4) | (M3) | (M4) | (M3) | (M4) | (M3) | (M4) |
| Parental BMI not measured (0-1) | -1.057 | -0.395 | -0.122 | -0.080 | -0.067 | -0.034 | -0.014 | -0.009 |
|  | (0.145) | (0.091) | (0.025) | (0.023) | (0.012) | (0.010) | (0.007) | (0.007) |
| At least one parent overweight (0-1) | -0.110 | -0.150 | -0.022 | -0.022 | -0.018 | -0.015 | -0.001 | -0.003 |
|  | (0.146) | (0.092) | (0.026) | (0.024) | (0.012) | (0.010) | (0.007) | (0.007) |
| At least one parent obese (0-1) | -0.707 | -0.308 | -0.045 | -0.027 | -0.044 | -0.025 | 0.009 | 0.010 |
|  | (0.146) | (0.093) | (0.026) | (0.024) | (0.012) | (0.010) | (0.008) | (0.007) |
| At least one parent smoker and/or heavy drinker (0-1) | 0.503 | 0.183 | 0.013 | -0.006 | 0.022 | 0.007 | 0.016 | 0.013 |
|  | (0.076) | (0.049) | (0.012) | (0.011) | (0.006) | (0.006) | (0.004) | (0.004) |
| Basic controls and GP fixed effects | Yes | Yes | Yes | Yes | Yes | Yes | Yes | Yes |
| Parental education and wages | Yes | Yes | Yes | Yes | Yes | Yes | Yes | Yes |
| Parental health care use | Yes | Yes | Yes | Yes | Yes | Yes | Yes | Yes |
| Parental health | Yes | Yes | Yes | Yes | Yes | Yes | Yes | Yes |
| Child health and lifestyle | - | Yes | - | Yes | - | Yes | - | Yes |
| Log. pseudolikelihood | -424914 | -298092 | -85251 | -79037 | -64362 | -58088 | -29911 | -29092 |
| Observations | 93365 | 93365 | 93365 | 93365 | 93365 | 93365 | 93365 | 93365 |

Notes: As Table A1.

**Table A4. Average marginal effects of basic controls and other regressors on their children’s type of health care use**

|  | Child GP visits | | | | Child specialist visits | | | | Child diagnostic tests | | | | Child ER visits | | | |
| --- | --- | --- | --- | --- | --- | --- | --- | --- | --- | --- | --- | --- | --- | --- | --- | --- |
|  | (M1) | (M2) | (M3) | (M4) | (M1) | (M2) | (M3) | (M4) | (M1) | (M2) | (M3) | (M4) | (M1) | (M2) | (M3) | (M4) |
| Girl (0-1) | -0.097 | 0.057 | -0.100 | 0.050 | -0.022 | -0.031 | -0.023 | -0.032 | 0.030 | 0.022 | 0.030 | 0.021 | -0.019 | -0.023 | -0.019 | -0.023 |
|  | (0.076) | (0.047) | (0.072) | (0.047) | (0.012) | (0.011) | (0.012) | (0.011) | (0.006) | (0.005) | (0.006) | (0.005) | (0.004) | (0.003) | (0.004) | (0.003) |
| 12-24 months (0-1) | 17.081 | 7.299 | 17.117 | 7.390 | 0.075 | 0.045 | 0.075 | 0.045 | 0.251 | 0.112 | 0.253 | 0.114 | 0.013 | 0.009 | 0.013 | 0.009 |
|  | (0.240) | (0.155) | (0.237) | (0.156) | (0.007) | (0.004) | (0.007) | (0.004) | (0.011) | (0.007) | (0.011) | (0.007) | (0.002) | (0.001) | (0.002) | (0.001) |
| 2-4 years (0-1) | 8.464 | 2.631 | 8.371 | 2.646 | 0.166 | 0.114 | 0.166 | 0.114 | 0.265 | 0.141 | 0.265 | 0.142 | 0.036 | 0.027 | 0.036 | 0.027 |
|  | (0.142) | (0.108) | (0.138) | (0.108) | (0.008) | (0.006) | (0.008) | (0.006) | (0.009) | (0.006) | (0.009) | (0.006) | (0.002) | (0.002) | (0.002) | (0.002) |
| 4-6 years (0-1) | 4.837 | 0.811 | 4.694 | 0.794 | 0.386 | 0.298 | 0.384 | 0.298 | 0.249 | 0.159 | 0.246 | 0.159 | 0.046 | 0.037 | 0.046 | 0.037 |
|  | (0.108) | (0.097) | (0.106) | (0.097) | (0.014) | (0.010) | (0.013) | (0.010) | (0.008) | (0.006) | (0.008) | (0.006) | (0.003) | (0.002) | (0.003) | (0.002) |
| 6-8 years (0-1) | 3.416 | 0.520 | 3.287 | 0.487 | 0.524 | 0.465 | 0.521 | 0.465 | 0.260 | 0.210 | 0.256 | 0.209 | 0.062 | 0.056 | 0.062 | 0.057 |
|  | (0.094) | (0.093) | (0.093) | (0.094) | (0.016) | (0.014) | (0.016) | (0.014) | (0.008) | (0.007) | (0.008) | (0.007) | (0.003) | (0.003) | (0.003) | (0.003) |
| 8-10 years (0-1) | 2.553 | 0.320 | 2.455 | 0.282 | 0.582 | 0.570 | 0.580 | 0.569 | 0.337 | 0.321 | 0.333 | 0.320 | 0.115 | 0.113 | 0.116 | 0.114 |
|  | (0.094) | (0.097) | (0.092) | (0.097) | (0.016) | (0.016) | (0.016) | (0.016) | (0.009) | (0.009) | (0.009) | (0.009) | (0.005) | (0.005) | (0.005) | (0.005) |
| 10-12 years (0-1) | 2.073 | 0.131 | 1.992 | 0.089 | 0.629 | 0.649 | 0.628 | 0.649 | 0.361 | 0.371 | 0.358 | 0.368 | 0.196 | 0.201 | 0.196 | 0.202 |
|  | (0.105) | (0.103) | (0.102) | (0.103) | (0.018) | (0.018) | (0.018) | (0.018) | (0.010) | (0.011) | (0.010) | (0.011) | (0.007) | (0.007) | (0.007) | (0.007) |
| 12-14 years (0-1) | 1.837 | 0.119 | 1.763 | 0.076 | 0.690 | 0.750 | 0.689 | 0.749 | 0.372 | 0.408 | 0.370 | 0.406 | 0.222 | 0.235 | 0.222 | 0.236 |
|  | (0.120) | (0.118) | (0.115) | (0.118) | (0.023) | (0.024) | (0.023) | (0.024) | (0.012) | (0.013) | (0.012) | (0.013) | (0.009) | (0.009) | (0.009) | (0.009) |
| 14-16 years (0-1) | 0.915 | -0.508 | 0.831 | -0.557 | 0.789 | 0.941 | 0.787 | 0.938 | 0.440 | 0.542 | 0.437 | 0.538 | 0.244 | 0.280 | 0.244 | 0.281 |
|  | (0.125) | (0.128) | (0.121) | (0.128) | (0.032) | (0.037) | (0.031) | (0.037) | (0.017) | (0.021) | (0.017) | (0.021) | (0.012) | (0.013) | (0.012) | (0.013) |
| 16-18 years (0-1) | 0.049 | -1.335 | -0.047 | -1.383 | 0.756 | 0.962 | 0.750 | 0.959 | 0.485 | 0.636 | 0.480 | 0.631 | 0.327 | 0.391 | 0.327 | 0.392 |
|  | (0.123) | (0.129) | (0.119) | (0.129) | (0.036) | (0.046) | (0.036) | (0.045) | (0.020) | (0.026) | (0.020) | (0.025) | (0.018) | (0.021) | (0.018) | (0.021) |
| Immigrant (0-1) | -1.065 | -0.233 | -1.022 | -0.213 | -0.107 | -0.069 | -0.099 | -0.069 | -0.037 | 0.003 | -0.031 | 0.006 | -0.028 | -0.021 | -0.026 | -0.020 |
|  | (0.465) | (0.391) | (0.463) | (0.391) | (0.053) | (0.051) | (0.054) | (0.051) | (0.035) | (0.032) | (0.035) | (0.032) | (0.021) | (0.022) | (0.022) | (0.022) |
| Fathers' age | -0.015 | 0.009 | -0.021 | 0.008 | 0.001 | 0.002 | 0.000 | 0.001 | -0.001 | 0.000 | -0.001 | 0.000 | -0.000 | -0.000 | -0.000 | -0.000 |
|  | (0.009) | (0.006) | (0.009) | (0.006) | (0.002) | (0.001) | (0.002) | (0.001) | (0.001) | (0.001) | (0.001) | (0.001) | (0.000) | (0.000) | (0.000) | (0.000) |
| Mothers' age | -0.005 | 0.003 | -0.012 | 0.003 | 0.001 | 0.001 | 0.000 | 0.001 | 0.000 | 0.000 | 0.000 | 0.000 | -0.000 | -0.000 | -0.001 | -0.000 |
|  | (0.009) | (0.006) | (0.009) | (0.006) | (0.002) | (0.001) | (0.002) | (0.001) | (0.001) | (0.001) | (0.001) | (0.001) | (0.000) | (0.000) | (0.000) | (0.000) |
| Min. distance to GP in km | -0.117 | -0.011 | -0.125 | -0.021 | 0.000 | 0.009 | 0.001 | 0.008 | -0.006 | 0.001 | -0.005 | 0.001 | 0.001 | 0.003 | 0.001 | 0.003 |
|  | (0.053) | (0.035) | (0.051) | (0.035) | (0.009) | (0.009) | (0.009) | (0.009) | (0.005) | (0.004) | (0.005) | (0.004) | (0.003) | (0.003) | (0.003) | (0.003) |
| Min. distance to Hospitals in km | 0.105 | 0.003 | 0.106 | 0.010 | -0.003 | -0.010 | -0.003 | -0.010 | 0.006 | -0.001 | 0.006 | -0.001 | -0.002 | -0.003 | -0.001 | -0.003 |
|  | (0.051) | (0.033) | (0.049) | (0.034) | (0.009) | (0.008) | (0.009) | (0.008) | (0.004) | (0.004) | (0.004) | (0.004) | (0.003) | (0.003) | (0.003) | (0.003) |
| Year=2005 (0-1) | 0.339 | 0.544 | 0.344 | 0.543 | 0.050 | 0.066 | 0.050 | 0.065 | -0.046 | -0.027 | -0.046 | -0.028 | -0.000 | 0.004 | -0.000 | 0.004 |
|  | (0.061) | (0.063) | (0.060) | (0.063) | (0.012) | (0.011) | (0.011) | (0.011) | (0.008) | (0.008) | (0.008) | (0.008) | (0.004) | (0.004) | (0.004) | (0.004) |
| Year=2006 (0-1) | 0.643 | 0.930 | 0.647 | 0.924 | 0.066 | 0.085 | 0.066 | 0.085 | -0.031 | -0.009 | -0.030 | -0.009 | 0.002 | 0.006 | 0.002 | 0.006 |
|  | (0.070) | (0.066) | (0.069) | (0.066) | (0.013) | (0.013) | (0.013) | (0.013) | (0.008) | (0.008) | (0.008) | (0.008) | (0.005) | (0.005) | (0.005) | (0.005) |
| Year=2007(0-1) | 0.784 | 1.423 | 0.805 | 1.418 | 0.036 | 0.065 | 0.037 | 0.064 | 0.000 | 0.035 | 0.001 | 0.035 | 0.004 | 0.011 | 0.004 | 0.011 |
|  | (0.079) | (0.069) | (0.078) | (0.069) | (0.014) | (0.013) | (0.014) | (0.013) | (0.009) | (0.009) | (0.009) | (0.009) | (0.005) | (0.005) | (0.005) | (0.005) |
| Year=2008 (0-1) | 1.126 | 1.863 | 1.174 | 1.869 | 0.032 | 0.065 | 0.032 | 0.064 | 0.032 | 0.074 | 0.034 | 0.074 | -0.003 | 0.005 | -0.003 | 0.004 |
|  | (0.086) | (0.076) | (0.085) | (0.076) | (0.015) | (0.014) | (0.015) | (0.014) | (0.010) | (0.009) | (0.010) | (0.009) | (0.005) | (0.005) | (0.005) | (0.005) |
| Year=2009 (0-1) | 1.688 | 1.975 | 1.754 | 2.000 | 0.143 | 0.142 | 0.143 | 0.142 | 0.026 | 0.038 | 0.028 | 0.038 | -0.010 | -0.008 | -0.010 | -0.009 |
|  | (0.095) | (0.082) | (0.094) | (0.081) | (0.017) | (0.016) | (0.017) | (0.016) | (0.010) | (0.009) | (0.010) | (0.009) | (0.005) | (0.005) | (0.005) | (0.005) |
| Year=2010 (0-1) | 1.200 | 1.744 | 1.295 | 1.781 | 0.137 | 0.167 | 0.138 | 0.167 | 0.024 | 0.057 | 0.027 | 0.058 | -0.002 | 0.003 | -0.002 | 0.002 |
|  | (0.098) | (0.079) | (0.097) | (0.079) | (0.018) | (0.018) | (0.018) | (0.018) | (0.011) | (0.010) | (0.011) | (0.010) | (0.006) | (0.006) | (0.006) | (0.006) |

**Table A4. Continued**

|  | Child GP visits | | | | Child specialist visits | | | | Child diagnostic tests | | | | Child ER visits | | | |
| --- | --- | --- | --- | --- | --- | --- | --- | --- | --- | --- | --- | --- | --- | --- | --- | --- |
|  | (M1) | (M2) | (M3) | (M4) | (M1) | (M2) | (M3) | (M4) | (M1) | (M2) | (M3) | (M4) | (M1) | (M2) | (M3) | (M4) |
| *Child health and lifestyle* |  |  |  |  |  |  |  |  |  |  |  |  |  |  |  |  |
| Children's number of comorbidities |  | 1.423 |  | 1.407 |  | 0.092 |  | 0.091 |  | 0.077 |  | 0.076 |  | 0.021 |  | 0.021 |
|  |  | (0.015) |  | (0.015) |  | (0.002) |  | (0.002) |  | (0.001) |  | (0.001) |  | (0.001) |  | (0.001) |
| Children with CVD (0-1) |  | 1.348 |  | 1.340 |  | 0.003 |  | 0.005 |  | 0.111 |  | 0.111 |  | 0.006 |  | 0.004 |
|  |  | (0.263) |  | (0.264) |  | (0.034) |  | (0.034) |  | (0.023) |  | (0.023) |  | (0.011) |  | (0.011) |
| Children with asthma/pulmonary problems (0-1) |  | 1.661 |  | 1.660 |  | 0.098 |  | 0.097 |  | 0.002 |  | 0.002 |  | 0.007 |  | 0.007 |
|  |  | (0.118) |  | (0.118) |  | (0.022) |  | (0.022) |  | (0.010) |  | (0.010) |  | (0.007) |  | (0.006) |
| Children with any mental disorder (0-1) |  | 0.311 |  | 0.351 |  | -0.081 |  | -0.080 |  | 0.007 |  | 0.008 |  | 0.002 |  | -0.001 |
|  |  | (0.451) |  | (0.450) |  | (0.055) |  | (0.056) |  | (0.032) |  | (0.031) |  | (0.022) |  | (0.021) |
| Child overweight (0-1) |  | 0.002 |  | 0.002 |  | 0.048 |  | 0.047 |  | -0.011 |  | -0.010 |  | 0.009 |  | 0.007 |
|  |  | (0.069) |  | (0.069) |  | (0.014) |  | (0.015) |  | (0.008) |  | (0.008) |  | (0.005) |  | (0.005) |
| Child obese (0-1) |  | 0.240 |  | 0.256 |  | 0.017 |  | 0.018 |  | -0.007 |  | -0.005 |  | 0.010 |  | 0.005 |
|  |  | (0.117) |  | (0.118) |  | (0.022) |  | (0.022) |  | (0.011) |  | (0.011) |  | (0.008) |  | (0.008) |
| Child BMI not available (0-1) |  | -4.896 |  | -4.844 |  | -0.290 |  | -0.285 |  | -0.198 |  | -0.193 |  | -0.014 |  | -0.014 |
|  |  | (0.068) |  | (0.069) |  | (0.013) |  | (0.013) |  | (0.008) |  | (0.008) |  | (0.005) |  | (0.005) |
| *Parental health* |  |  |  |  |  |  |  |  |  |  |  |  |  |  |  |  |
| Parental number of comorbidities |  |  | 0.359 | 0.090 |  |  | 0.020 | 0.003 |  |  | 0.019 | 0.005 |  |  | 0.000 | -0.003 |
|  |  |  | (0.014) | (0.009) |  |  | (0.002) | (0.002) |  |  | (0.001) | (0.001) |  |  | (0.001) | (0.001) |
| Fathers' hospitalization days |  |  | -0.037 | 0.001 |  |  | -0.004 | -0.003 |  |  | -0.002 | -0.001 |  |  | -0.002 | -0.002 |
|  |  |  | (0.018) | (0.011) |  |  | (0.003) | (0.003) |  |  | (0.001) | (0.001) |  |  | (0.001) | (0.001) |
| Mothers' hospitalization days |  |  | -0.066 | -0.032 |  |  | 0.002 | 0.004 |  |  | -0.003 | -0.001 |  |  | 0.000 | 0.001 |
|  |  |  | (0.021) | (0.015) |  |  | (0.005) | (0.004) |  |  | (0.002) | (0.002) |  |  | (0.001) | (0.001) |
| Any parent with mental disorder (0-1) |  |  | -0.312 | -0.138 |  |  | -0.025 | -0.016 |  |  | -0.006 | 0.002 |  |  | -0.003 | -0.002 |
|  |  |  | (0.092) | (0.061) |  |  | (0.015) | (0.014) |  |  | (0.008) | (0.007) |  |  | (0.004) | (0.004) |
| Any parent passed away (0-1) |  |  | 0.261 | -0.194 |  |  | -0.018 | -0.063 |  |  | 0.003 | -0.031 |  |  | 0.029 | 0.013 |
|  |  |  | (0.305) | (0.203) |  |  | (0.050) | (0.046) |  |  | (0.028) | (0.024) |  |  | (0.018) | (0.013) |

**Table A4. Continued**

|  | Child GP visits | | | | Child specialist visits | | | | Child diagnostic tests | | | | Child ER visits | | | |
| --- | --- | --- | --- | --- | --- | --- | --- | --- | --- | --- | --- | --- | --- | --- | --- | --- |
|  | (M1) | (M2) | (M3) | (M4) | (M1) | (M2) | (M3) | (M4) | (M1) | (M2) | (M3) | (M4) | (M1) | (M2) | (M3) | (M4) |
| GP fixed effects | Yes | Yes | Yes | Yes | Yes | Yes | Yes | Yes | Yes | Yes | Yes | Yes | Yes | Yes | Yes | Yes |
| Parental education and wages | Yes | Yes | Yes | Yes | Yes | Yes | Yes | Yes | Yes | Yes | Yes | Yes | Yes | Yes | Yes | Yes |
| Parental health care use | Yes | Yes | Yes | Yes | Yes | Yes | Yes | Yes | Yes | Yes | Yes | Yes | Yes | Yes | Yes | Yes |
| Parental lifestyle | - | - | Yes | Yes | - | - | Yes | Yes | - | - | Yes | Yes | - | - | Yes | Yes |
| Log. pseudolikelihood | -434538 | -298753 | -424914 | -298092 | -85823 | -79138 | -85251 | -79037 | -64894 | -58143 | -64362 | -58088 | -29978 | -29146 | -29911 | -29092 |
| Observations | 93365 | 93365 | 93365 | 93365 | 93365 | 93365 | 93365 | 93365 | 93365 | 93365 | 93365 | 93365 | 93365 | 93365 | 93365 | 93365 |

Notes: As Table A1.

**Table A5. Average marginal effects of parental health care use on their children’s type of health care use – with child fixed effects**

|  | Child GP visits | | | | Child specialist visits | | | | Child diagnostic tests | | | | Child ER visits | | | |
| --- | --- | --- | --- | --- | --- | --- | --- | --- | --- | --- | --- | --- | --- | --- | --- | --- |
|  | (M1) | (M2) | (M3) | (M4) | (M1) | (M2) | (M3) | (M4) | (M1) | (M2) | (M3) | (M4) | (M1) | (M2) | (M3) | (M4) |
| Fathers' visits to GP | 0.151 | 0.066 | 0.063 | 0.022 | -0.001 | -0.006 | -0.007 | -0.009 | 0.008 | 0.003 | 0.002 | 0.001 | -0.002 | -0.004 | -0.001 | -0.002 |
|  | (0.021) | (0.016) | (0.023) | (0.017) | (0.006) | (0.007) | (0.007) | (0.007) | (0.003) | (0.003) | (0.004) | (0.004) | (0.004) | (0.004) | (0.005) | (0.005) |
| Mothers' visits to GP | 0.174 | 0.112 | 0.092 | 0.071 | 0.002 | -0.004 | -0.004 | -0.007 | 0.006 | -0.002 | -0.001 | -0.004 | 0.004 | 0.003 | 0.006 | 0.006 |
|  | (0.017) | (0.013) | (0.018) | (0.015) | (0.006) | (0.006) | (0.006) | (0.006) | (0.003) | (0.003) | (0.003) | (0.003) | (0.004) | (0.004) | (0.004) | (0.004) |
| Fathers' visits to specialist | 0.087 | -0.005 | 0.083 | -0.010 | 0.012 | 0.008 | 0.009 | 0.007 | 0.005 | -0.002 | 0.003 | -0.002 | 0.004 | 0.003 | 0.008 | 0.007 |
|  | (0.041) | (0.030) | (0.042) | (0.031) | (0.013) | (0.013) | (0.014) | (0.014) | (0.007) | (0.007) | (0.008) | (0.007) | (0.009) | (0.009) | (0.009) | (0.009) |
| Mothers' visits to specialist | -0.057 | -0.045 | -0.064 | -0.058 | 0.024 | 0.023 | 0.027 | 0.026 | 0.005 | 0.004 | 0.008 | 0.005 | 0.010 | 0.010 | 0.009 | 0.007 |
|  | (0.035) | (0.026) | (0.036) | (0.027) | (0.009) | (0.009) | (0.009) | (0.009) | (0.005) | (0.005) | (0.006) | (0.006) | (0.008) | (0.008) | (0.008) | (0.008) |
| Fathers' urgency visits | -0.420 | -0.293 | -0.407 | -0.292 | 0.010 | 0.000 | 0.007 | -0.003 | -0.033 | -0.017 | -0.031 | -0.016 | 0.049 | 0.064 | 0.058 | 0.072 |
|  | (0.161) | (0.135) | (0.162) | (0.137) | (0.042) | (0.046) | (0.042) | (0.046) | (0.025) | (0.027) | (0.025) | (0.027) | (0.025) | (0.025) | (0.025) | (0.026) |
| Mothers' urgency visits | -0.536 | -0.413 | -0.581 | -0.448 | 0.004 | 0.015 | 0.007 | 0.018 | -0.055 | -0.044 | -0.050 | -0.037 | -0.009 | -0.011 | -0.014 | -0.016 |
|  | (0.159) | (0.125) | (0.162) | (0.127) | (0.048) | (0.048) | (0.049) | (0.048) | (0.024) | (0.024) | (0.024) | (0.024) | (0.024) | (0.026) | (0.024) | (0.026) |
| Basic controls and individual fixed effects | Yes | Yes | Yes | Yes | Yes | Yes | Yes | Yes | Yes | Yes | Yes | Yes | Yes | Yes | Yes | Yes |
| Child health and lifestyle | - | Yes | - | Yes | - | Yes | - | Yes | - | Yes | - | Yes | - | Yes | - | Yes |
| Parental health and lifestyle | - | - | Yes | Yes | - | - | Yes | Yes | - | - | Yes | Yes | - | - | Yes | Yes |
| Log. pseudolikelihood | -342945 | -258588 | -342346 | -258441 | -54754 | -52943 | -54742 | -52935 | -48066 | -44774 | -48045 | -44765 | -19642 | -19391 | -19635 | -19383 |
| Absorbed fixed effects | 13852 | 13852 | 13852 | 13852 | 7547 | 7547 | 7547 | 7547 | 8754 | 8754 | 8754 | 8754 | 4718 | 4718 | 4718 | 4718 |
| Observations | 88319 | 88319 | 88319 | 88319 | 47677 | 47677 | 47677 | 47677 | 55724 | 55724 | 55724 | 55724 | 28487 | 28487 | 28487 | 28487 |

Notes: Marginal effects from four Poisson model specifications that include child fixed effects. Specifications 1-4 are designated as M1-M4. Robust standard errors in parentheses. Observations that are either singletons or separated by a fixed effect are dropped from the estimation sample. Only time-varying covariates are included, except for child age. However, all models include dummies for every year and individual fixed effects (which include cohort effects) and, thereby, control for age effects (these three variables are linearly dependent: age = year - cohort). Each set of included variables is described in Table 1.

**Table A6. Average marginal effects of other included regressors on their children’s type of health care use – with child fixed effects**

|  | Child GP visits | | | | Child specialist visits | | | | Child diagnostic tests | | | | Child ER visits | | | |
| --- | --- | --- | --- | --- | --- | --- | --- | --- | --- | --- | --- | --- | --- | --- | --- | --- |
|  | (M1) | (M2) | (M3) | (M4) | (M1) | (M2) | (M3) | (M4) | (M1) | (M2) | (M3) | (M4) | (M1) | (M2) | (M3) | (M4) |
| *Basic controls* |  |  |  |  |  |  |  |  |  |  |  |  |  |  |  |  |
| Year=2005 (0-1) | 0.367 | 0.441 | 0.372 | 0.446 | 0.127 | 0.154 | 0.127 | 0.154 | -0.038 | -0.005 | -0.038 | -0.005 | 0.031 | 0.039 | 0.031 | 0.039 |
|  | (0.084) | (0.066) | (0.084) | (0.066) | (0.018) | (0.017) | (0.018) | (0.017) | (0.011) | (0.009) | (0.011) | (0.009) | (0.010) | (0.009) | (0.010) | (0.009) |
| Year=2006 (0-1) | 0.599 | 0.660 | 0.606 | 0.661 | 0.217 | 0.277 | 0.218 | 0.277 | 0.014 | 0.061 | 0.014 | 0.061 | 0.085 | 0.098 | 0.085 | 0.098 |
|  | (0.083) | (0.066) | (0.083) | (0.065) | (0.020) | (0.019) | (0.020) | (0.019) | (0.012) | (0.010) | (0.011) | (0.010) | (0.011) | (0.011) | (0.011) | (0.011) |
| Year=2007 (0-1) | 0.619 | 0.990 | 0.630 | 0.995 | 0.233 | 0.338 | 0.234 | 0.338 | 0.101 | 0.191 | 0.102 | 0.190 | 0.146 | 0.172 | 0.148 | 0.173 |
|  | (0.084) | (0.067) | (0.084) | (0.067) | (0.020) | (0.020) | (0.020) | (0.020) | (0.012) | (0.012) | (0.012) | (0.012) | (0.012) | (0.012) | (0.012) | (0.012) |
| Year=2008 (0-1) | 0.799 | 1.307 | 0.814 | 1.317 | 0.308 | 0.443 | 0.309 | 0.443 | 0.202 | 0.327 | 0.202 | 0.328 | 0.190 | 0.229 | 0.191 | 0.230 |
|  | (0.087) | (0.070) | (0.086) | (0.070) | (0.021) | (0.021) | (0.021) | (0.021) | (0.014) | (0.014) | (0.014) | (0.014) | (0.013) | (0.014) | (0.013) | (0.014) |
| Year=2009 (0-1) | 1.156 | 1.224 | 1.175 | 1.236 | 0.665 | 0.798 | 0.665 | 0.798 | 0.239 | 0.331 | 0.240 | 0.331 | 0.239 | 0.266 | 0.240 | 0.267 |
|  | (0.091) | (0.070) | (0.091) | (0.070) | (0.025) | (0.025) | (0.025) | (0.025) | (0.014) | (0.014) | (0.014) | (0.014) | (0.015) | (0.015) | (0.015) | (0.015) |
| Year=2010 (0-1) | 0.434 | 0.925 | 0.467 | 0.943 | 0.783 | 1.063 | 0.785 | 1.064 | 0.284 | 0.468 | 0.285 | 0.466 | 0.367 | 0.429 | 0.370 | 0.433 |
|  | (0.096) | (0.074) | (0.096) | (0.074) | (0.029) | (0.032) | (0.029) | (0.032) | (0.015) | (0.016) | (0.015) | (0.016) | (0.018) | (0.020) | (0.018) | (0.020) |
| Fathers' age | 0.418 | 0.258 | 0.421 | 0.256 | 0.019 | 0.001 | 0.020 | 0.002 | 0.017 | 0.005 | 0.016 | 0.004 | 0.001 | 0.000 | 0.001 | 0.000 |
|  | (0.047) | (0.031) | (0.046) | (0.030) | (0.011) | (0.012) | (0.011) | (0.012) | (0.006) | (0.005) | (0.006) | (0.005) | (0.007) | (0.007) | (0.007) | (0.007) |
| Mothers' age | 0.793 | 0.397 | 0.803 | 0.402 | 0.089 | 0.071 | 0.089 | 0.071 | 0.043 | 0.022 | 0.045 | 0.023 | 0.014 | 0.006 | 0.014 | 0.007 |
|  | (0.053) | (0.032) | (0.053) | (0.032) | (0.015) | (0.014) | (0.015) | (0.014) | (0.007) | (0.006) | (0.007) | (0.006) | (0.008) | (0.008) | (0.008) | (0.008) |
| *Child health and lifestyle* |  |  |  |  |  |  |  |  |  |  |  |  |  |  |  |  |
| Children's number of comorbidities |  | 1.656 |  | 1.653 |  | 0.140 |  | 0.140 |  | 0.129 |  | 0.128 |  | 0.050 |  | 0.051 |
|  |  | (0.011) |  | (0.011) |  | (0.003) |  | (0.003) |  | (0.002) |  | (0.002) |  | (0.002) |  | (0.002) |
| Children with CVD (0-1) |  | -0.805 |  | -0.805 |  | 0.040 |  | 0.040 |  | -0.115 |  | -0.115 |  | 0.093 |  | 0.096 |
|  |  | (0.358) |  | (0.358) |  | (0.120) |  | (0.120) |  | (0.043) |  | (0.043) |  | (0.080) |  | (0.080) |
| Children with asthma/pulmonary problems (0-1) |  | -0.116 |  | -0.117 |  | 0.116 |  | 0.117 |  | 0.025 |  | 0.024 |  | -0.009 |  | -0.009 |
|  |  | (0.180) |  | (0.180) |  | (0.073) |  | (0.073) |  | (0.038) |  | (0.038) |  | (0.042) |  | (0.042) |
| Children with any mental disorder (0-1) |  | 0.233 |  | 0.232 |  | -0.080 |  | -0.079 |  | -0.061 |  | -0.060 |  | -0.089 |  | -0.089 |
|  |  | (0.513) |  | (0.511) |  | (0.157) |  | (0.157) |  | (0.080) |  | (0.080) |  | (0.067) |  | (0.067) |
| Child overweight (0-1) |  | -0.964 |  | -0.957 |  | 0.188 |  | 0.189 |  | 0.016 |  | 0.016 |  | 0.029 |  | 0.028 |
|  |  | (0.083) |  | (0.083) |  | (0.030) |  | (0.030) |  | (0.017) |  | (0.017) |  | (0.017) |  | (0.017) |
| Child obese (0-1) |  | -1.571 |  | -1.571 |  | 0.306 |  | 0.305 |  | 0.016 |  | 0.017 |  | 0.024 |  | 0.020 |
|  |  | (0.142) |  | (0.142) |  | (0.062) |  | (0.062) |  | (0.032) |  | (0.032) |  | (0.034) |  | (0.034) |
| Child BMI not available (0-1) |  | -6.932 |  | -6.912 |  | 0.012 |  | 0.007 |  | -0.221 |  | -0.223 |  | 0.063 |  | 0.068 |
|  |  | (0.138) |  | (0.139) |  | (0.224) |  | (0.221) |  | (0.071) |  | (0.070) |  | (0.118) |  | (0.120) |

**Table A6. Continued**

|  | Child GP visits | | | | Child specialist visits | | | | Child diagnostic tests | | | | Child ER visits | | | |
| --- | --- | --- | --- | --- | --- | --- | --- | --- | --- | --- | --- | --- | --- | --- | --- | --- |
|  | (M1) | (M2) | (M3) | (M4) | (M1) | (MM2) | (M3) | (M4) | (M1) | (M2) | (M3) | (M4) | (M1) | (M2) | (M3) | (M4) |
| *Parental health and lifestyle* |  |  |  |  |  |  |  |  |  |  |  |  |  |  |  |  |
| Parental number of comorbidities |  |  | 0.412 | 0.214 |  |  | 0.032 | 0.016 |  |  | 0.031 | 0.011 |  |  | -0.009 | -0.014 |
|  |  |  | (0.041) | (0.034) |  |  | (0.015) | (0.015) |  |  | (0.007) | (0.007) |  |  | (0.010) | (0.010) |
| Fathers' hospitalization days |  |  | -0.026 | 0.047 |  |  | 0.012 | 0.015 |  |  | -0.005 | -0.003 |  |  | -0.057 | -0.050 |
|  |  |  | (0.088) | (0.047) |  |  | (0.014) | (0.013) |  |  | (0.008) | (0.007) |  |  | (0.049) | (0.051) |
| Mothers' hospitalization days |  |  | -0.053 | 0.190 |  |  | -0.055 | -0.061 |  |  | -0.059 | -0.051 |  |  | 0.023 | 0.031 |
|  |  |  | (0.142) | (0.122) |  |  | (0.063) | (0.064) |  |  | (0.039) | (0.046) |  |  | (0.018) | (0.018) |
| Any parent with mental disorder (0-1) |  |  | -0.907 | -0.155 |  |  | -0.152 | -0.076 |  |  | -0.076 | -0.006 |  |  | 0.067 | 0.118 |
|  |  |  | (0.638) | (0.469) |  |  | (0.209) | (0.186) |  |  | (0.103) | (0.102) |  |  | (0.136) | (0.159) |
| Any parent passed away (0-1) |  |  | -0.482 | -0.571 |  |  | -0.355 | -0.394 |  |  | 1.403 | 0.448 |  |  |  |  |
|  |  |  | (7.152) | (3.235) |  |  | (0.389) | (0.415) |  |  | (1.214) | (0.580) |  |  |  |  |
| Parental BMI not measured (0-1) |  |  | 2.978 | 1.503 |  |  | 0.090 | 0.102 |  |  | 0.073 | 0.027 |  |  | 0.112 | 0.083 |
|  |  |  | (0.471) | (0.380) |  |  | (0.119) | (0.121) |  |  | (0.061) | (0.061) |  |  | (0.096) | (0.097) |
| At least one parent overweight (0-1) |  |  | 1.310 | 1.129 |  |  | 0.187 | 0.205 |  |  | 0.165 | 0.161 |  |  | -0.064 | -0.087 |
|  |  |  | (0.462) | (0.393) |  |  | (0.135) | (0.135) |  |  | (0.070) | (0.070) |  |  | (0.089) | (0.092) |
| At least one parent obese (0-1) |  |  | 1.631 | 1.147 |  |  | 0.168 | 0.143 |  |  | 0.220 | 0.206 |  |  | 0.138 | 0.113 |
|  |  |  | (0.549) | (0.472) |  |  | (0.165) | (0.154) |  |  | (0.088) | (0.090) |  |  | (0.118) | (0.119) |
| At least one parent smoker and/or heavy drinker (0-1) |  |  | -0.281 | 0.225 |  |  | -0.336 | -0.337 |  |  | -0.036 | 0.063 |  |  | -0.113 | -0.096 |
|  |  |  | (0.580) | (0.446) |  |  | (0.162) | (0.147) |  |  | (0.080) | (0.071) |  |  | (0.097) | (0.096) |
| Individual fixed effects | Yes | Yes | Yes | Yes | Yes | Yes | Yes | Yes | Yes | Yes | Yes | Yes | Yes | Yes | Yes | Yes |
| Parental health care use | Yes | Yes | Yes | Yes | Yes | Yes | Yes | Yes | Yes | Yes | Yes | Yes | Yes | Yes | Yes | Yes |
| Log. pseudolikelihood | -342945 | -258588 | -342346 | -258441 | -54754 | -52943 | -54742 | -52935 | -48066 | -44774 | -48045 | -44765 | -19642 | -19391 | -19635 | -19383 |
| Absorbed fixed effects | 13852 | 13852 | 13852 | 13852 | 7547 | 7547 | 7547 | 7547 | 8754 | 8754 | 8754 | 8754 | 4718 | 4718 | 4718 | 4718 |
| Observations | 88319 | 88319 | 88319 | 88319 | 47677 | 47677 | 47677 | 47677 | 55724 | 55724 | 55724 | 55724 | 28487 | 28487 | 28487 | 28487 |

Notes: As Table A5.

**Table A7. Average marginal effects of parental education and household labor income on their daughters’ and sons’ type of health care use**

|  | Child GP visits | | | | Child specialist visits | | | | Child diagnostic tests | | | | Child ER visits | | | |
| --- | --- | --- | --- | --- | --- | --- | --- | --- | --- | --- | --- | --- | --- | --- | --- | --- |
| **Daughters** | (M1) | (M2) | (M3) | (M4) | (M1) | (M2) | (M3) | (M4) | (M1) | (M2) | (M3) | (M4) | (M1) | (M2) | (M3) | (M4) |
| Father secondary (0-1) | 0.136 | -0.032 | -0.018 | -0.066 | 0.054 | 0.046 | 0.046 | 0.044 | 0.016 | 0.008 | 0.008 | 0.006 | -0.013 | -0.013 | -0.011 | -0.011 |
|  | (0.148) | (0.088) | (0.143) | (0.089) | (0.022) | (0.020) | (0.022) | (0.021) | (0.013) | (0.011) | (0.013) | (0.011) | (0.007) | (0.007) | (0.007) | (0.007) |
| Father tertiary (0-1) | -0.379 | -0.062 | -0.372 | -0.077 | -0.026 | -0.008 | -0.027 | -0.011 | -0.015 | 0.004 | -0.016 | 0.002 | -0.032 | -0.028 | -0.028 | -0.025 |
|  | (0.241) | (0.134) | (0.226) | (0.134) | (0.033) | (0.033) | (0.033) | (0.033) | (0.019) | (0.018) | (0.019) | (0.018) | (0.010) | (0.010) | (0.010) | (0.010) |
| Mother secondary (0-1) | 0.482 | 0.076 | 0.414 | 0.066 | -0.002 | 0.001 | 0.002 | 0.002 | 0.039 | 0.035 | 0.040 | 0.035 | -0.010 | -0.008 | -0.008 | -0.006 |
|  | (0.151) | (0.094) | (0.147) | (0.095) | (0.024) | (0.022) | (0.024) | (0.022) | (0.012) | (0.011) | (0.012) | (0.011) | (0.007) | (0.007) | (0.007) | (0.006) |
| Mother tertiary (0-1) | 0.020 | 0.037 | 0.118 | 0.054 | -0.023 | 0.019 | -0.009 | 0.022 | 0.025 | 0.051 | 0.037 | 0.054 | -0.014 | -0.005 | -0.008 | -0.001 |
|  | (0.214) | (0.131) | (0.206) | (0.131) | (0.034) | (0.034) | (0.035) | (0.034) | (0.019) | (0.017) | (0.019) | (0.017) | (0.010) | (0.010) | (0.010) | (0.010) |
| Inferred parental salaries | -0.011 | -0.004 | -0.009 | -0.004 | -0.002 | -0.001 | -0.001 | -0.001 | -0.001 | -0.001 | -0.001 | -0.001 | -0.000 | -0.000 | -0.000 | -0.000 |
|  | (0.003) | (0.002) | (0.003) | (0.002) | (0.000) | (0.000) | (0.000) | (0.000) | (0.000) | (0.000) | (0.000) | (0.000) | (0.000) | (0.000) | (0.000) | (0.000) |
| Log. pseudolikelihood | -209100 | -143553 | -203991 | -143238 | -40546 | -37385 | -40295 | -37341 | -32244 | -28819 | -31949 | -28790 | -13710 | -13270 | -13657 | -13226 |
| Observations | 45174 | 45174 | 45174 | 45174 | 45174 | 45174 | 45174 | 45174 | 45174 | 45174 | 45174 | 45174 | 45174 | 45174 | 45174 | 45174 |
| **Sons** | (M1) | (M2) | (M3) | (M4) | (M1) | (M2) | (M3) | (M4) | (M1) | (M2) | (M3) | (M4) | (M1) | (M2) | (M3) | (M4) |
| Father secondary (0-1) | 0.330 | 0.113 | 0.191 | 0.064 | 0.007 | 0.004 | 0.001 | 0.003 | 0.016 | 0.012 | 0.011 | 0.011 | -0.015 | -0.013 | -0.014 | -0.011 |
|  | (0.148) | (0.097) | (0.147) | (0.098) | (0.022) | (0.020) | (0.022) | (0.020) | (0.011) | (0.010) | (0.011) | (0.010) | (0.007) | (0.007) | (0.007) | (0.007) |
| Father tertiary (0-1) | -0.298 | -0.102 | -0.228 | -0.099 | -0.049 | -0.016 | -0.043 | -0.014 | -0.007 | 0.008 | -0.003 | 0.009 | -0.031 | -0.024 | -0.028 | -0.022 |
|  | (0.221) | (0.140) | (0.217) | (0.142) | (0.033) | (0.032) | (0.034) | (0.032) | (0.017) | (0.016) | (0.017) | (0.016) | (0.010) | (0.009) | (0.010) | (0.009) |
| Mother secondary (0-1) | 0.288 | -0.005 | 0.242 | -0.015 | 0.013 | 0.008 | 0.021 | 0.012 | 0.004 | -0.004 | 0.006 | -0.003 | 0.008 | 0.006 | 0.009 | 0.006 |
|  | (0.156) | (0.100) | (0.150) | (0.100) | (0.022) | (0.020) | (0.021) | (0.020) | (0.012) | (0.010) | (0.011) | (0.010) | (0.007) | (0.006) | (0.007) | (0.006) |
| Mother tertiary (0-1) | 0.001 | -0.000 | 0.145 | 0.032 | 0.015 | 0.045 | 0.043 | 0.057 | -0.000 | 0.017 | 0.014 | 0.021 | 0.014 | 0.015 | 0.018 | 0.017 |
|  | (0.217) | (0.135) | (0.208) | (0.135) | (0.034) | (0.032) | (0.034) | (0.032) | (0.017) | (0.015) | (0.017) | (0.015) | (0.010) | (0.010) | (0.010) | (0.010) |
| Inferred parental salaries | -0.018 | -0.006 | -0.018 | -0.007 | -0.002 | -0.001 | -0.002 | -0.001 | -0.001 | -0.000 | -0.001 | -0.000 | -0.000 | -0.000 | -0.000 | -0.000 |
|  | (0.003) | (0.002) | (0.003) | (0.002) | (0.000) | (0.000) | (0.000) | (0.000) | (0.000) | (0.000) | (0.000) | (0.000) | (0.000) | (0.000) | (0.000) | (0.000) |
| Log. pseudolikelihood | -224358 | -154768 | -219851 | -154417 | -44851 | -41346 | -44512 | -41260 | -32464 | -29173 | -32229 | -29141 | -16095 | -15709 | -16066 | -15688 |
| Observations | 48191 | 48191 | 48191 | 48191 | 48191 | 48191 | 48191 | 48191 | 48191 | 48191 | 48191 | 48191 | 48191 | 48191 | 48191 | 48191 |
| Basic controls and GP fixed effects | Yes | Yes | Yes | Yes | Yes | Yes | Yes | Yes | Yes | Yes | Yes | Yes | Yes | Yes | Yes | Yes |
| Parental health care use | Yes | Yes | Yes | Yes | Yes | Yes | Yes | Yes | Yes | Yes | Yes | Yes | Yes | Yes | Yes | Yes |
| Child health and lifestyle | - | Yes | - | Yes | - | Yes | - | Yes | - | Yes | - | Yes | - | Yes | - | Yes |
| Parental health and lifestyle | - | - | Yes | Yes | - | - | Yes | Yes | - | - | Yes | Yes | - | - | Yes | Yes |

Notes: same notes as Table A1; models estimated here separately for girls and boys.

**Table A8. Average marginal effects of parental health care use on their daughters’ and sons’ type of health care use**

|  | Child GP visits | | | | Child specialist visits | | | | Child diagnostic tests | | | | Child ER visits | | | |
| --- | --- | --- | --- | --- | --- | --- | --- | --- | --- | --- | --- | --- | --- | --- | --- | --- |
| **Daughters** | (M1) | (M2) | (M3) | (M4) | (M1) | (M2) | (M3) | (M4) | (M1) | (M2) | (M3) | (M4) | (M1) | (M2) | (M3) | (M4) |
| Fathers' visits to GP | 0.044 | 0.011 | -0.012 | -0.001 | 0.002 | 0.000 | -0.000 | 0.000 | 0.003 | 0.001 | -0.000 | 0.000 | 0.000 | 0.000 | 0.000 | 0.000 |
|  | (0.007) | (0.004) | (0.008) | (0.005) | (0.001) | (0.001) | (0.001) | (0.001) | (0.001) | (0.000) | (0.001) | (0.001) | (0.000) | (0.000) | (0.001) | (0.001) |
| Mothers' visits to GP | 0.082 | 0.029 | 0.041 | 0.018 | 0.005 | 0.001 | 0.003 | 0.001 | 0.004 | 0.001 | 0.001 | -0.000 | 0.000 | -0.000 | 0.000 | -0.000 |
|  | (0.010) | (0.004) | (0.006) | (0.004) | (0.001) | (0.001) | (0.001) | (0.001) | (0.001) | (0.000) | (0.000) | (0.000) | (0.000) | (0.000) | (0.000) | (0.000) |
| Fathers' visits to specialist | 0.044 | 0.014 | 0.007 | 0.002 | 0.009 | 0.006 | 0.007 | 0.006 | 0.004 | 0.003 | 0.002 | 0.002 | 0.001 | 0.000 | 0.001 | 0.001 |
|  | (0.014) | (0.008) | (0.013) | (0.009) | (0.002) | (0.002) | (0.002) | (0.002) | (0.001) | (0.001) | (0.001) | (0.001) | (0.001) | (0.001) | (0.001) | (0.001) |
| Mothers' visits to specialist | 0.066 | 0.002 | 0.046 | 0.002 | 0.012 | 0.010 | 0.010 | 0.009 | 0.005 | 0.002 | 0.003 | 0.002 | 0.001 | 0.001 | 0.001 | 0.001 |
|  | (0.013) | (0.007) | (0.012) | (0.007) | (0.002) | (0.001) | (0.002) | (0.002) | (0.001) | (0.001) | (0.001) | (0.001) | (0.000) | (0.000) | (0.001) | (0.000) |
| Fathers' urgency visits | 0.019 | 0.026 | -0.041 | 0.007 | -0.002 | -0.001 | -0.000 | 0.002 | -0.003 | -0.002 | -0.005 | -0.002 | 0.013 | 0.014 | 0.014 | 0.015 |
|  | (0.057) | (0.035) | (0.053) | (0.037) | (0.008) | (0.007) | (0.008) | (0.008) | (0.005) | (0.004) | (0.005) | (0.004) | (0.002) | (0.002) | (0.002) | (0.002) |
| Mothers' urgency visits | 0.047 | 0.000 | -0.047 | -0.009 | 0.012 | 0.009 | 0.008 | 0.008 | 0.012 | 0.008 | 0.007 | 0.007 | 0.018 | 0.017 | 0.018 | 0.017 |
|  | (0.058) | (0.035) | (0.053) | (0.035) | (0.008) | (0.008) | (0.008) | (0.008) | (0.004) | (0.004) | (0.004) | (0.004) | (0.002) | (0.002) | (0.002) | (0.002) |
| Log. pseudolikelihood | -209100 | -143553 | -203991 | -143238 | -40546 | -37385 | -40295 | -37341 | -32244 | -28819 | -31949 | -28790 | -13710 | -13270 | -13657 | -13226 |
| Observations | 45174 | 45174 | 45174 | 45174 | 45174 | 45174 | 45174 | 45174 | 45174 | 45174 | 45174 | 45174 | 45174 | 45174 | 45174 | 45174 |
| **Sons** | (M1) | (M2) | (M3) | (M4) | (M1) | (M2) | (M3) | (M4) | (M1) | (M2) | (M3) | (M4) | (M1) | (M2) | (M3) | (M4) |
| Fathers' visits to GP | 0.037 | 0.009 | -0.005 | -0.004 | 0.002 | -0.001 | -0.003 | -0.003 | 0.001 | -0.001 | -0.001 | -0.002 | -0.000 | -0.000 | -0.000 | -0.000 |
|  | (0.009) | (0.004) | (0.007) | (0.005) | (0.001) | (0.001) | (0.001) | (0.001) | (0.000) | (0.000) | (0.001) | (0.001) | (0.000) | (0.000) | (0.000) | (0.000) |
| Mothers' visits to GP | 0.097 | 0.035 | 0.052 | 0.022 | 0.006 | 0.001 | 0.002 | 0.000 | 0.004 | 0.001 | 0.002 | 0.000 | 0.000 | -0.000 | 0.000 | -0.000 |
|  | (0.008) | (0.004) | (0.007) | (0.005) | (0.001) | (0.001) | (0.001) | (0.001) | (0.001) | (0.000) | (0.001) | (0.000) | (0.000) | (0.000) | (0.000) | (0.000) |
| Fathers' visits to specialist | 0.039 | 0.003 | 0.010 | -0.002 | 0.009 | 0.007 | 0.007 | 0.006 | 0.003 | 0.001 | 0.002 | 0.001 | 0.000 | -0.000 | 0.000 | 0.000 |
|  | (0.012) | (0.008) | (0.013) | (0.008) | (0.002) | (0.001) | (0.002) | (0.002) | (0.001) | (0.001) | (0.001) | (0.001) | (0.001) | (0.001) | (0.001) | (0.001) |
| Mothers' visits to specialist | 0.052 | 0.010 | 0.035 | 0.007 | 0.009 | 0.008 | 0.008 | 0.007 | 0.005 | 0.003 | 0.004 | 0.003 | 0.001 | 0.001 | 0.001 | 0.001 |
|  | (0.011) | (0.008) | (0.010) | (0.008) | (0.002) | (0.001) | (0.002) | (0.001) | (0.001) | (0.001) | (0.001) | (0.001) | (0.001) | (0.000) | (0.001) | (0.001) |
| Fathers' urgency visits | 0.126 | 0.090 | 0.035 | 0.064 | 0.006 | 0.005 | 0.004 | 0.006 | 0.004 | 0.002 | 0.001 | 0.002 | 0.022 | 0.021 | 0.022 | 0.022 |
|  | (0.056) | (0.038) | (0.054) | (0.039) | (0.009) | (0.008) | (0.009) | (0.009) | (0.004) | (0.004) | (0.004) | (0.004) | (0.003) | (0.002) | (0.003) | (0.002) |
| Mothers' urgency visits | -0.119 | -0.128 | -0.174 | -0.134 | -0.011 | -0.015 | -0.014 | -0.016 | -0.002 | -0.003 | -0.004 | -0.004 | 0.018 | 0.019 | 0.019 | 0.019 |
|  | (0.061) | (0.039) | (0.054) | (0.039) | (0.008) | (0.008) | (0.008) | (0.008) | (0.004) | (0.004) | (0.004) | (0.004) | (0.003) | (0.002) | (0.003) | (0.002) |
| Log. pseudolikelihood | -224358 | -154768 | -219851 | -154417 | -44851 | -41346 | -44512 | -41260 | -32464 | -29173 | -32229 | -29141 | -16095 | -15709 | -16066 | -15688 |
| Observations | 48191 | 48191 | 48191 | 48191 | 48191 | 48191 | 48191 | 48191 | 48191 | 48191 | 48191 | 48191 | 48191 | 48191 | 48191 | 48191 |
| Basic controls and GP fixed effects | Yes | Yes | Yes | Yes | Yes | Yes | Yes | Yes | Yes | Yes | Yes | Yes | Yes | Yes | Yes | Yes |
| Parental education and wages | Yes | Yes | Yes | Yes | Yes | Yes | Yes | Yes | Yes | Yes | Yes | Yes | Yes | Yes | Yes | Yes |
| Child health and lifestyle | - | Yes | - | Yes | - | Yes | - | Yes | - | Yes | - | Yes | - | Yes | - | Yes |
| Parental health and lifestyle | - | - | Yes | Yes | - | - | Yes | Yes | - | - | Yes | Yes | - | - | Yes | Yes |

Notes: same notes as Table A1; models estimated here separately for girls and boys.

**Table A9. Average marginal effects of parental health care use on their daughters’ and sons’ type of health care use – with child fixed effects**

|  | Child GP visits | | | | Child specialist visits | | | | Child diagnostic tests | | | | Child ER visits | | | |
| --- | --- | --- | --- | --- | --- | --- | --- | --- | --- | --- | --- | --- | --- | --- | --- | --- |
| **Daughters** | (M1) | (M2) | (M3) | (M4) | (M1) | (M2) | (M3) | (M4) | (M1) | (M2) | (M3) | (M4) | (M1) | (M2) | (M3) | (M4) |
| Fathers' visits to GP | 0.145 | 0.058 | 0.015 | -0.012 | -0.002 | -0.004 | -0.011 | -0.011 | 0.007 | 0.002 | -0.002 | -0.003 | -0.005 | -0.007 | -0.007 | -0.008 |
|  | (0.031) | (0.025) | (0.035) | (0.028) | (0.008) | (0.008) | (0.009) | (0.009) | (0.005) | (0.004) | (0.005) | (0.005) | (0.008) | (0.008) | (0.009) | (0.008) |
| Mothers' visits to GP | 0.169 | 0.108 | 0.056 | 0.048 | 0.001 | -0.002 | -0.005 | -0.006 | 0.008 | 0.000 | -0.003 | -0.005 | 0.008 | 0.008 | 0.007 | 0.007 |
|  | (0.024) | (0.018) | (0.026) | (0.021) | (0.008) | (0.007) | (0.008) | (0.008) | (0.005) | (0.004) | (0.005) | (0.005) | (0.006) | (0.006) | (0.007) | (0.006) |
| Fathers' visits to specialist | 0.134 | 0.022 | 0.107 | 0.000 | -0.028 | -0.028 | -0.041 | -0.042 | -0.005 | -0.015 | -0.008 | -0.019 | 0.038 | 0.037 | 0.036 | 0.035 |
|  | (0.056) | (0.041) | (0.054) | (0.044) | (0.018) | (0.019) | (0.019) | (0.020) | (0.011) | (0.011) | (0.011) | (0.011) | (0.012) | (0.012) | (0.012) | (0.012) |
| Mothers' visits to specialist | 0.010 | -0.020 | 0.009 | -0.017 | 0.024 | 0.020 | 0.026 | 0.021 | 0.001 | -0.003 | -0.001 | -0.004 | 0.011 | 0.009 | 0.016 | 0.013 |
|  | (0.046) | (0.033) | (0.048) | (0.034) | (0.011) | (0.011) | (0.011) | (0.011) | (0.007) | (0.006) | (0.008) | (0.007) | (0.013) | (0.013) | (0.014) | (0.013) |
| Fathers' urgency visits | -0.454 | -0.294 | -0.463 | -0.307 | 0.053 | 0.031 | 0.050 | 0.026 | -0.026 | -0.001 | -0.025 | -0.002 | -0.027 | -0.013 | -0.027 | -0.014 |
|  | (0.242) | (0.194) | (0.246) | (0.199) | (0.056) | (0.064) | (0.057) | (0.064) | (0.035) | (0.035) | (0.036) | (0.035) | (0.041) | (0.041) | (0.042) | (0.042) |
| Mothers' urgency visits | -0.429 | -0.371 | -0.480 | -0.385 | -0.027 | -0.017 | -0.037 | -0.022 | -0.012 | 0.017 | -0.015 | 0.016 | -0.109 | -0.130 | -0.106 | -0.127 |
|  | (0.247) | (0.198) | (0.252) | (0.200) | (0.073) | (0.076) | (0.075) | (0.078) | (0.039) | (0.036) | (0.040) | (0.037) | (0.046) | (0.044) | (0.046) | (0.044) |
| Log. pseudolikelihood | -162414 | -124378 | -161855 | -124195 | -25926 | -25126 | -25910 | -25114 | -24015 | -22359 | -23992 | -22347 | -8761 | -8633 | -8755 | -8629 |
| Absorbed fixed effects | 6684 | 6684 | 6684 | 6684 | 3644 | 3644 | 3644 | 3644 | 4339 | 4339 | 4339 | 4339 | 2139 | 2139 | 2139 | 2139 |
| Observations | 42562 | 42562 | 42562 | 42562 | 22988 | 22988 | 22988 | 22988 | 27546 | 27546 | 27546 | 27546 | 12821 | 12821 | 12821 | 12821 |
| **Sons** | (M1) | (M2) | (M3) | (M4) | (M1) | (M2) | (M3) | (M4) | (M1) | (M2) | (M3) | (M4) | (M1) | (M2) | (M3) | (M4) |
| Fathers' visits to GP | 0.157 | 0.072 | 0.101 | 0.047 | -0.001 | -0.008 | -0.004 | -0.008 | 0.010 | 0.006 | 0.007 | 0.006 | 0.000 | -0.002 | 0.002 | 0.001 |
|  | (0.029) | (0.021) | (0.031) | (0.023) | (0.010) | (0.010) | (0.011) | (0.012) | (0.005) | (0.005) | (0.005) | (0.005) | (0.005) | (0.005) | (0.006) | (0.006) |
| Mothers' visits to GP | 0.176 | 0.116 | 0.123 | 0.092 | 0.003 | -0.006 | -0.001 | -0.005 | 0.004 | -0.004 | 0.001 | -0.004 | 0.002 | 0.001 | 0.006 | 0.006 |
|  | (0.023) | (0.018) | (0.025) | (0.020) | (0.008) | (0.008) | (0.010) | (0.011) | (0.003) | (0.004) | (0.004) | (0.005) | (0.005) | (0.005) | (0.005) | (0.005) |
| Fathers' visits to specialist | 0.030 | -0.034 | 0.038 | -0.031 | 0.050 | 0.040 | 0.050 | 0.042 | 0.014 | 0.010 | 0.014 | 0.011 | -0.024 | -0.028 | -0.015 | -0.019 |
|  | (0.071) | (0.042) | (0.073) | (0.043) | (0.017) | (0.017) | (0.018) | (0.018) | (0.010) | (0.010) | (0.011) | (0.010) | (0.014) | (0.014) | (0.014) | (0.014) |
| Mothers' visits to specialist | -0.119 | -0.070 | -0.128 | -0.090 | 0.024 | 0.027 | 0.035 | 0.037 | 0.012 | 0.015 | 0.020 | 0.020 | 0.009 | 0.008 | 0.006 | 0.004 |
|  | (0.050) | (0.040) | (0.051) | (0.042) | (0.014) | (0.014) | (0.015) | (0.015) | (0.008) | (0.008) | (0.008) | (0.009) | (0.009) | (0.010) | (0.009) | (0.009) |
| Fathers' urgency visits | -0.404 | -0.298 | -0.375 | -0.288 | -0.029 | -0.025 | -0.026 | -0.022 | -0.037 | -0.029 | -0.035 | -0.025 | 0.090 | 0.104 | 0.104 | 0.116 |
|  | (0.216) | (0.188) | (0.218) | (0.190) | (0.061) | (0.064) | (0.062) | (0.065) | (0.035) | (0.040) | (0.035) | (0.041) | (0.027) | (0.027) | (0.028) | (0.028) |
| Mothers' urgency visits | -0.558 | -0.420 | -0.609 | -0.479 | 0.018 | 0.025 | 0.015 | 0.019 | -0.084 | -0.087 | -0.075 | -0.073 | 0.026 | 0.031 | 0.022 | 0.025 |
|  | (0.208) | (0.161) | (0.213) | (0.164) | (0.066) | (0.062) | (0.068) | (0.064) | (0.030) | (0.033) | (0.031) | (0.033) | (0.027) | (0.027) | (0.027) | (0.027) |
| Log. pseudolikelihood | -180421 | -134141 | -180198 | -134082 | -28806 | -27793 | -28800 | -27786 | -24036 | -22389 | -24027 | -22383 | -10864 | -10735 | -10859 | -10728 |
| Absorbed fixed effects | 7168 | 7168 | 7168 | 7168 | 3903 | 3903 | 3903 | 3903 | 4415 | 4415 | 4415 | 4415 | 2579 | 2579 | 2579 | 2579 |
| Observations | 45757 | 45757 | 45757 | 45757 | 24689 | 24689 | 24689 | 24689 | 28178 | 28178 | 28178 | 28178 | 15666 | 15666 | 15666 | 15666 |
| Basic controls and individual fixed effects | Yes | Yes | Yes | Yes | Yes | Yes | Yes | Yes | Yes | Yes | Yes | Yes | Yes | Yes | Yes | Yes |
| Child health and lifestyle | - | Yes | - | Yes | - | Yes | - | Yes | - | Yes | - | Yes | - | Yes | - | Yes |
| Parental health and lifestyle | - | - | Yes | Yes | - | - | Yes | Yes | - | - | Yes | Yes | - | - | Yes | Yes |

Notes: same notes as Table A5; models estimated here separately for girls and boys.

**Table A10. Unadjusted p-values and FDR-adjusted q-values for parental health care utilization coefficients in Panels C and D in Figure 2**

| Group | Outcome | X-var | p_value | q_BH |
| --- | --- | --- | --- | --- |
| Children | Diagnostic tests | Fathers' ER visits | 0.5600 | 0.7740 |
| Children | Diagnostic tests | Fathers' visits to GP | 0.7748 | 0.8800 |
| Children | Diagnostic tests | Fathers' visits to specialist | 0.7984 | 0.8889 |
| Children | Diagnostic tests | Mothers' ER visits | 0.1288 | 0.3534 |
| Children | Diagnostic tests | Mothers' visits to GP | 0.2035 | 0.4742 |
| Children | Diagnostic tests | Mothers' visits to specialist | 0.3267 | 0.5992 |
| Children | Emergency room (ER) visits | Fathers' ER visits | 0.0045 | 0.0362 |
| Children | Emergency room (ER) visits | Fathers' visits to GP | 0.6204 | 0.8067 |
| Children | Emergency room (ER) visits | Fathers' visits to specialist | 0.4778 | 0.7544 |
| Children | Emergency room (ER) visits | Mothers' ER visits | 0.5251 | 0.7544 |
| Children | Emergency room (ER) visits | Mothers' visits to GP | 0.1708 | 0.4431 |
| Children | Emergency room (ER) visits | Mothers' visits to specialist | 0.3786 | 0.6608 |
| Children | GP visits | Fathers' ER visits | 0.0325 | 0.1189 |
| Children | GP visits | Fathers' visits to GP | 0.2105 | 0.4742 |
| Children | GP visits | Fathers' visits to specialist | 0.7421 | 0.8796 |
| Children | GP visits | Mothers' ER visits | 0.0004 | 0.0049 |
| Children | GP visits | Mothers' visits to GP | 0.0000 | 0.0000 |
| Children | GP visits | Mothers' visits to specialist | 0.0322 | 0.1189 |
| Children | Specialist visits | Fathers' ER visits | 0.9472 | 0.9571 |
| Children | Specialist visits | Fathers' visits to GP | 0.2225 | 0.4853 |
| Children | Specialist visits | Fathers' visits to specialist | 0.6218 | 0.8067 |
| Children | Specialist visits | Mothers' ER visits | 0.7141 | 0.8569 |
| Children | Specialist visits | Mothers' visits to GP | 0.2547 | 0.5316 |
| Children | Specialist visits | Mothers' visits to specialist | 0.0049 | 0.0363 |

Notes: q-values are computed using the Benjamini–Hochberg false discovery rate (FDR) procedure applied jointly to the parental health care utilization coefficients (father’s and mother’s GP visits, specialist visits, and emergency visits) across all outcomes and model specifications.

**Table A11. Unadjusted p-values and FDR-adjusted q-values for parental health care utilization coefficients in Panels C and D in Figures 5 and 6**

| Group | Outcome | X_var | p_value | q_BH |
| --- | --- | --- | --- | --- |
| Boys | Diagnostic tests | Fathers' ER visits | 0.5353 | 0.7039 |
| Boys | Diagnostic tests | Fathers' visits to GP | 0.2568 | 0.4825 |
| Boys | Diagnostic tests | Fathers' visits to specialist | 0.2706 | 0.4849 |
| Boys | Diagnostic tests | Mothers' ER visits | 0.0274 | 0.0908 |
| Boys | Diagnostic tests | Mothers' visits to GP | 0.4008 | 0.6055 |
| Boys | Diagnostic tests | Mothers' visits to specialist | 0.0178 | 0.0647 |
| Boys | Emergency room (ER) visits | Fathers' ER visits | 0.0000 | 0.0007 |
| Boys | Emergency room (ER) visits | Fathers' visits to GP | 0.9004 | 0.9294 |
| Boys | Emergency room (ER) visits | Fathers' visits to specialist | 0.1715 | 0.3688 |
| Boys | Emergency room (ER) visits | Mothers' ER visits | 0.3580 | 0.5544 |
| Boys | Emergency room (ER) visits | Mothers' visits to GP | 0.2803 | 0.4892 |
| Boys | Emergency room (ER) visits | Mothers' visits to specialist | 0.6762 | 0.8140 |
| Boys | GP visits | Fathers' ER visits | 0.1308 | 0.2951 |
| Boys | GP visits | Fathers' visits to GP | 0.0374 | 0.1159 |
| Boys | GP visits | Fathers' visits to specialist | 0.4654 | 0.6571 |
| Boys | GP visits | Mothers' ER visits | 0.0036 | 0.0268 |
| Boys | GP visits | Mothers' visits to GP | 0.0000 | 0.0001 |
| Boys | GP visits | Mothers' visits to specialist | 0.0313 | 0.1003 |
| Boys | Specialist visits | Fathers' ER visits | 0.7341 | 0.8101 |
| Boys | Specialist visits | Fathers' visits to GP | 0.5125 | 0.6833 |
| Boys | Specialist visits | Fathers' visits to specialist | 0.0182 | 0.0628 |
| Boys | Specialist visits | Mothers' ER visits | 0.7666 | 0.8363 |
| Boys | Specialist visits | Mothers' visits to GP | 0.6155 | 0.7879 |
| Boys | Specialist visits | Mothers' visits to specialist | 0.0119 | 0.0571 |
| Girls | Diagnostic tests | Fathers' ER visits | 0.9563 | 0.9767 |
| Girls | Diagnostic tests | Fathers' visits to GP | 0.5516 | 0.8682 |
| Girls | Diagnostic tests | Fathers' visits to specialist | 0.0964 | 0.3084 |
| Girls | Diagnostic tests | Mothers' ER visits | 0.6480 | 0.8640 |
| Girls | Diagnostic tests | Mothers' visits to GP | 0.3039 | 0.6641 |
| Girls | Diagnostic tests | Mothers' visits to specialist | 0.5665 | 0.8705 |
| Girls | Emergency room (ER) visits | Fathers' ER visits | 0.7401 | 0.8772 |
| Girls | Emergency room (ER) visits | Fathers' visits to GP | 0.3156 | 0.6586 |
| Girls | Emergency room (ER) visits | Fathers' visits to specialist | 0.0037 | 0.0411 |
| Girls | Emergency room (ER) visits | Mothers' ER visits | 0.0039 | 0.0411 |
| Girls | Emergency room (ER) visits | Mothers' visits to GP | 0.2829 | 0.6466 |
| Girls | Emergency room (ER) visits | Mothers' visits to specialist | 0.3044 | 0.6587 |
| Girls | GP visits | Fathers' ER visits | 0.1231 | 0.3694 |
| Girls | GP visits | Fathers' visits to GP | 0.6753 | 0.8530 |
| Girls | GP visits | Fathers' visits to specialist | 0.9986 | 0.9986 |
| Girls | GP visits | Mothers' ER visits | 0.0538 | 0.2348 |
| Girls | GP visits | Mothers' visits to GP | 0.0228 | 0.1459 |
| Girls | GP visits | Mothers' visits to specialist | 0.6165 | 0.8878 |
| Girls | Specialist visits | Fathers' ER visits | 0.6901 | 0.8604 |
| Girls | Specialist visits | Fathers' visits to GP | 0.2253 | 0.5407 |
| Girls | Specialist visits | Fathers' visits to specialist | 0.0348 | 0.1758 |
| Girls | Specialist visits | Mothers' ER visits | 0.7777 | 0.8888 |
| Girls | Specialist visits | Mothers' visits to GP | 0.4503 | 0.8005 |
| Girls | Specialist visits | Mothers' visits to specialist | 0.0424 | 0.2034 |

Notes: q-values are computed using the Benjamini–Hochberg false discovery rate (FDR) procedure applied jointly to the parental health care utilization coefficients (father’s and mother’s GP visits, specialist visits, and emergency visits) across all outcomes and model specifications.
